# Quantifying Magnetic Anisotropy of Ferroelectric Fe(II) Square‐Pyramidal Systems Using Torque Magnetometry

**DOI:** 10.1002/advs.75759

**Published:** 2026-05-20

**Authors:** Vijaya Thangaraj, Gemma K. Gransbury, Deepanshu Chauhan, Dipanti Borah, Gopalan Rajaraman, Mauro Perfetti, Maheswaran Shanmugam

**Affiliations:** ^1^ Department of Chemistry Indian Institute of Technology Bombay Mumbai Maharashtra India; ^2^ Dipartimento di Chimica “Ugo Schiff” and UdR INSTM Università degli Studi di Firenze Sesto Fiorentino Italy

**Keywords:** cantilever torque magnetometry, coordination complex, ferroelectric, iron

## Abstract

The design of mononuclear complexes that simultaneously exhibit multiple functionalities represents a rapidly advancing frontier in molecular materials research. Achieving strong easy‐axis magnetic anisotropy within inherently polar crystal lattices remains a significant challenge, yet it offers a promising pathway toward next‐generation spin–electric materials. Comparative studies of isostructural systems with subtle structural variations provide key insight into molecular tuning strategies for practical implementation. In this context, we report a family of five‐coordinate, square‐pyramidal Fe(II) complexes, [Fe(L)(X)_2_]·CHCl_3_ (L = tridentate Schiff‐base ligand; X = Cl (**1**), Br (**2**)), crystallizing in the polar, non‐centrosymmetric triclinic space group *P1* and exhibiting pronounced easy‐axis magnetic anisotropy. Cantilever torque magnetometry on single crystals reveals a negative axial Zero Field Splitting parameter *D* = −25.6 cm^−^^1^ (**1**) and −19.8 cm^−^^1^ (**2**), among the largest reported for square‐pyramidal Fe(II). Ab initio CASSCF/NEVPT2 calculations reproduce the experimental Spin Hamiltonian parameters and show that subtle steric and electronic effects, particularly the out‐of‐plane displacement of Fe(II), critically govern the magnitude and sign of *D*. Complementary piezoresponse force microscopy and Polarization vs Electric field measurements confirm intrinsic polarization and a pronounced nanoscale piezoelectric response consistent with computed dipole moments, establishing a compelling platform for multifunctional spin–electric materials and multifunctional molecular architectures.

## Graphical abstract

Five‐coordinate, square‐pyramidal Fe(II) complexes adopting polar, non‐centrosymmetric crystal structures exhibit exceptionally large easy‐axis magnetic anisotropy. Ligand‐driven distortions critically control the anisotropy, and the dielectric, Polarization vs Elecric field and piezo response studies confirm intrinsic polarization, positioning these complexes as a rare class of multifunctional molecular materials with strong prospects for spin–electric coupling.

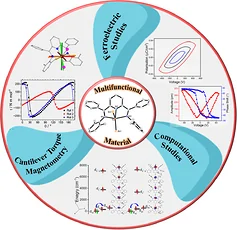

## 1 Introduction

The relentless miniaturization of data storage and information technologies has brought conventional magnetic materials to their intrinsic limits, triggering the search for molecular‐scale storage units in which individual molecules act as bistable bits. Central to this endeavor is the realization of single‐molecule magnets: molecules capable of retaining their magnetization for a sufficiently long period of time [1, 2]. To push blocking temperatures (*T_B_*) and relaxation times higher, one must maximize the effective energy barrier (*U_eff_*), which depends on both a high‐spin ground state and strong axial magnetic anisotropy in transition metal complexes. Early efforts using polynuclear transition‐metal clusters achieved high spin multiplicities, but noncollinear orientation among local anisotropy axes often led to cancellation effects that limited *U_eff_* [3, 4, 5]. This motivated a shift toward mononuclear single‐ion magnets (SIMs), where the ligand field and molecular symmetry can be precisely controlled to modulate the magnetic anisotropy. Although the most performant SIMs are currently based on lanthanide ions [6, 7, 8], earth‐abundant transition metals‐based systems have shown impressive results: two‐coordinate Co(II) complexes with bulky N‐heterocyclic carbene ligands have achieved record anisotropy barriers (∼ 413–450 cm^−^^1^), while a linear Fe(I) complex, [Fe(C(SiMe_3_)_3_)_2_]^−^, achieved *U*_*eff*_ ≈ 226 cm^−^^1^, firmly establishing that iron can be competitive [9, 10]. Although these reported complexes have provided valuable insights into the factors governing spin–orbit coupling and, consequently, magnetic anisotropy, their poor stability under ambient conditions severely restricts their practical applicability. Achieving robust easy‐axis magnetic anisotropy while maintaining long‐term air stability remains a formidable challenge. While systematic investigations and, to some extent, magneto‐structural correlations have been extensively developed for Co(II) systems across various coordination geometries [11, 12, 13, 14, 15], comparable in‐depth studies are notably scarce for Fe(II) complexes, particularly those adopting a square‐pyramidal geometry. Metal ions with substantial spin–orbit coupling often exhibit large Zero‐Field Splitting (ZFS), frequently exceeding the energy window accessible to conventional spectroscopic techniques such as *X*‐ and *Q*‐band EPR. Nonetheless, accurate determination of the electronic structure remains essential. Advanced methods, including high‐frequency EPR (HF‐EPR), inelastic neutron scattering (INS), magnetic circular dichroism (MCD), and far‐infrared (FIR) spectroscopy, can yield detailed ZFS parameters, but they demand highly specialized instrumentation and favorable sample characteristics [16, 17]. In contrast, cantilever torque magnetometry (CTM), performed on oriented single crystals across wide temperature and magnetic‐field ranges, enables precise extraction of Spin Hamiltonian (SH) parameters (including the *g*‐ and *D*‐tensors) [18]. Although CTM also carries inherent limitations that can complicate data interpretation, it remains a powerful and accessible alternative for probing magnetic anisotropy. Beyond magnetic properties, the presence of a non‐centrosymmetric (polar) lattice is an added advantage in constructing multifunctionality: the absence of inversion symmetry allows intrinsic polarization, which is required for piezoelectricity, ferroelectricity, nonlinear optical properties, and spin–electric coupling (SEC) [19, 20, 21]. Recent work has demonstrated chemical tuning of SEC in molecular magnets, showing how modest structural changes and built‐in electric dipoles in lanthanide or transition metal complexes can allow electric field modulation of ZFS or spin states [22]. Nonetheless, combining robust magnetic anisotropy with non‐centrosymmetric lattices in a transition‐metal complex remains rare, because it requires fine‐tuned coordination geometry, ligand design, and supramolecular interactions.

In this context, we report here air‐stable Fe(II) systems that crystallize in a polar space group with large easy‐axis magnetic anisotropy compared to other Fe(II) five‐coordinate square‐pyramidal complexes, with *D* values of −25.6 cm^−^^1^ (**1**) and −19.8 cm^−^^1^ (**2**), as unambiguously determined by CTM and fully corroborated by detailed DC magnetic studies and ab initio CASSCF/NEVPT2 calculations. Dielectric measurements reveal intrinsic polarization, while both lateral and vertical piezo force microscopy (PFM) studies on polycrystalline samples of **1** and **2,** and Polarization vs Electric field measurements clearly demonstrate nanoscale piezoelectric and ferroelectric responses. Furthermore, ab initio calculations performed facilitate understanding the electronic structure of the complexes and the rationale for the sign and magnitude of the observed *D* values in these complexes. These complexes nonetheless represent a rare convergence of pronounced axial anisotropy, persistent polarization, and local ferro‐/piezoelectric functionality within a single Fe(II) molecular platform.

## 2 Results and Discussion

**SCHEME 1:**
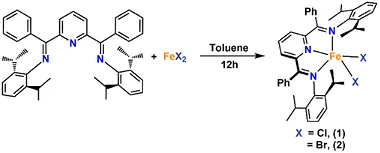
A general synthetic scheme was followed to isolate complexes **1** and **2**.

**FIGURE 1:**
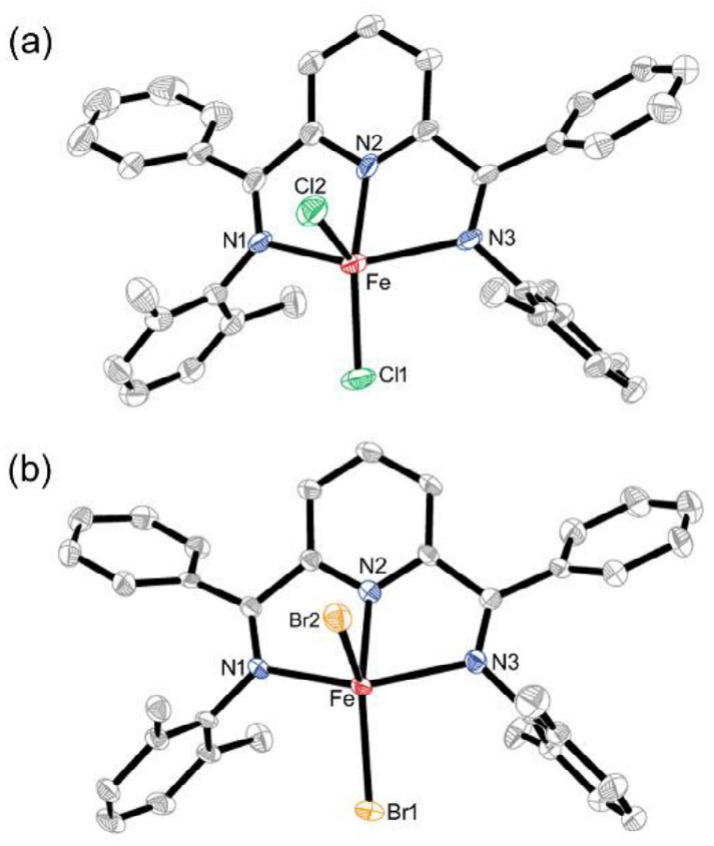
(a, b) Thermal ellipsoid (50% probability) representation of the molecular structure of **1** and **2**. Hydrogen atoms, side chain carbon atoms, and solvent molecules are removed for clarity.

The reaction of a dry toluene solution of 2,6‐bis{1‐[(2,6‐diisopropylphenyl)imino]benzyl}pyridine (L) with toluene suspensions of anhydrous FeX_2_ (X = Cl, Br) upon heating/stirring produces a green colored precipitate in the reaction mixture. Recrystallization of this residue in chloroform/hexane yields block‐shaped green crystals within one day (Scheme 1). The molecular structures of both complexes were determined using single‐crystal X‐ray diffraction, revealing their molecular formulae as [Fe(L)(X_2_)]·CHCl_3_ (where X = Cl (**1**) or Br (**2**); Figure 1). Both complexes crystallize in the triclinic, polar *P1* space group, with one complete metal complex and a chloroform molecule in the entire unit cell (Table S1). Single‐crystal X‐ray diffraction (SCXRD) analysis reveals that the two complexes are isostructural; the detailed structural descriptions of **1** and **2** are detailed below. The structure solution reveals that **1** is a mononuclear Fe(II) complex, whose coordination sites were completed by the tridentate pincer ligand L and two chloride ions (bromides in case of **2**; Figure 1). Thus, the metal center in **1** (as well as in **2**) adopts a distorted square pyramidal geometry, which was confirmed by Continuous Shape Measurement software (CShM), with CShM values of 2.45 and 2.94 for **1** and **2**, respectively. We recall that a non‐zero CShM value indicates the extent of deviation from the ideal square pyramidal geometry (Table S2).

In the equatorial plane there are three nitrogen atoms (from L) and one halide ion, and the axial position is occupied by the second halide ion. In both cases, the Fe(II) ion does not occupy the center of the average mean plane of these atoms, but it is shifted out‐of‐plane by 0.54 Å (**1**) and 0.52 Å (**2**). Such an out‐of‐plane shift has a significant role in determining not only the sign of *D* (axial zero field splitting parameter) but also its magnitude on several complexes, including the recent example reported by some of us [13, 23] (Figure S1).

The average Fe–N bond distance is 2.149(3) Å. Notably, the axial halide bond length is longer (Fe–‐Cl_ax_ = 2.351(7) (**1**), Fe–Br_ax_ = 2.502(4) Å (**2**)) than the equatorial bond length (Fe–‐Cl_eq_ = 2.246(16) (**1**), Fe–‐Br_eq_ = 2.383(4) Å (**2**)). The selected bond distances and bond angles of **1** and **2** are detailed in Table S3 of the ESI.

**FIGURE 2:**
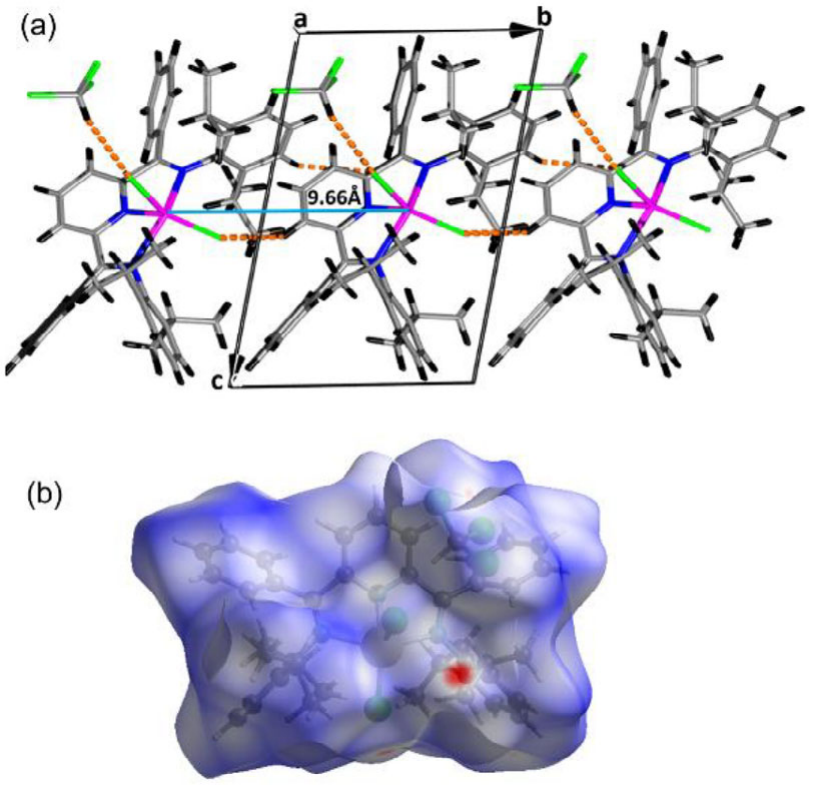
(a) Packing diagram of **1** along the *a*‐axis. The dotted orange lines represent intermolecular hydrogen bonding. (b) Hirshfeld surface view of complex **1**.

The halide ion and the CHCl_3_ solvent molecule in the crystal lattice of **1** and **2** are involved in intermolecular H‐bonding interactions with the phenyl rings of L, resulting in the construction of a supramolecular network (Figure 2a and Figure S2). Hirshfeld surface analysis was carried out to quantify the supramolecular interactions present within the crystal lattice. The analysis reveals that Cl···H and C···H contacts contribute 13.3% and 7.3%, respectively, to the overall intermolecular interactions, alongside other contacts within the lattice (Figure 2b and Figure S3).

The PXRD profiles of **1** and **2** (Figure S4) were in good agreement with the simulation, indicating the bulk phase purity of the isolated complexes.

Further, to gain insight into the electronic structure and to elucidate both the sign and magnitude of the *D* parameter for these complexes, HF‐EPR measurements were carried out at 5 K, but were unsuccessful.

Consequently, to unambiguously determine both the sign and magnitude of the zero‐field splitting parameters for complexes **1** and **2**, cantilever torque magnetometry (CTM) was employed.

### 2.1 Cantilever Torque Magnetometry (CTM)

With crystallographically equivalent molecules, the *P1* space group provides the possibility to experimentally map magnetic anisotropy without relying on theoretical inputs. Leveraging on this structural advantage, we employed CTM to determine the magnetic anisotropy tensors of complexes **1** and **2**. Although many iron complexes are known, their ZFS parameters have rarely been quantitatively evaluated, making this analysis particularly significant [18]. An indexed single crystal was mounted on a piezoelectric cantilever torque magnetometer, and three rotations were performed along three perpendicular axes in the 2–270 K temperature range with magnetic fields up to 9 T (Table S4 and Figures S5, S6) [24]. The resulting torque curves and static magnetic data (Figures S7–S12 and Figures 4, 5) were simultaneously fit to a unified model in EasySpin [25] with the optimization performed using *esfit* (weighting procedure detailed in ESI). The torque was calculated as the negative derivative of the free energy with respect to the rotation angle [18]. The model used *S* = 2, rhombic zero‐field splitting (ZFS), rhombic *g* values, and a scaling parameter to account for uncertainty in the mass of the crystal (see equation 1 and ESI for details). Importantly, the *g* and ZFS reference frames were fitted independently here for the first time on CTM data. Fitted parameters are given in Table 1.

**TABLE 1:** Spin Hamiltonian parameters extracted from CTM and CASSCF/NEVPT2 calculations for complexes **1** and **2**.

Complex	*g*_x_	*g*_y_	*g*_z_	*D* (cm^−1^)	*E*/*D*	Method
1	1.909(9)	2.311(8)	2.530(3)	−25.6(4)	0.208(8)	CTM
	1.902	2.247	2.652	−29.87	0.316	NEVPT2
2	2.010(4)	2.279(3)	2.536(2)	−19.8(2)	0.144(5)	CTM
	1.925	2.120	2.628	−29.78	0.232	NEVPT2

The principal axes of the ZFS tensor are precisely defined by the low‐temperature, high‐field torque curves (Figure 4). In both **1** and **2** the ZFS easy axis is closest to the Fe–X bond in the equatorial plane, which was also observed for Co(II) analogues with Cl^−^, Br^−^ and I^−^ [13].

**FIGURE 3:**
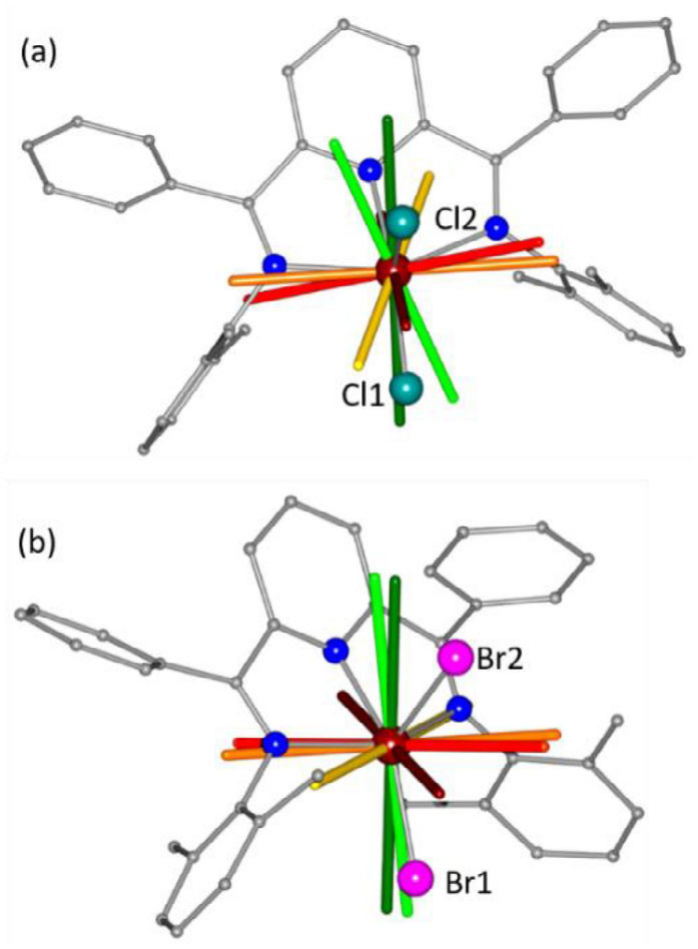
(a, b) Experimental principal directions for *g* and *D* for **1** and **2,** respectively. Vector color code: *D*_z_ easy axis (dark green), *D*_y_ intermediate axis (orange), *D*_x_ hard axis (maroon), *g*_z_ easy axis (light green), *g*_y_ intermediate axis (yellow), *g*_x_ hard axis (red).

The ZFS intermediate axis is most closely aligned to one of the Fe imine N bonds, while the hard axis does not align with any structural features but is close to the FeCl_2_ plane in **1** (Figure 3).

On increasing temperature, the ZFS levels are more evenly populated, and the resulting reduced *D* anisotropy is observed as a reduction in the magnitude of the torque signal (Figures S8 and S11). At higher temperatures, the torque signal is dominated by *g*‐anisotropy, although with the large magnitude of ZFS, both *g* and *D* anisotropy contribute to the signal at room temperature. In **2**, the *g*_z_ and *D*_z_ easy axes are closely aligned, with *g*_z_ also aligned with Fe–Br_1_. On the contrary, in **1** the *g* and *D* easy axes are more tilted (22.5°) with respect to each other, and *g*_z_ is more offset from Fe–Cl_1_ (Figures 3; Figures S13–S18 and Table S5). The intermediate and hard axes are not conserved between the *g* and *D* frames, neither for **1** nor **2**, this is primarily observed in rotation 2.

**FIGURE 4:**
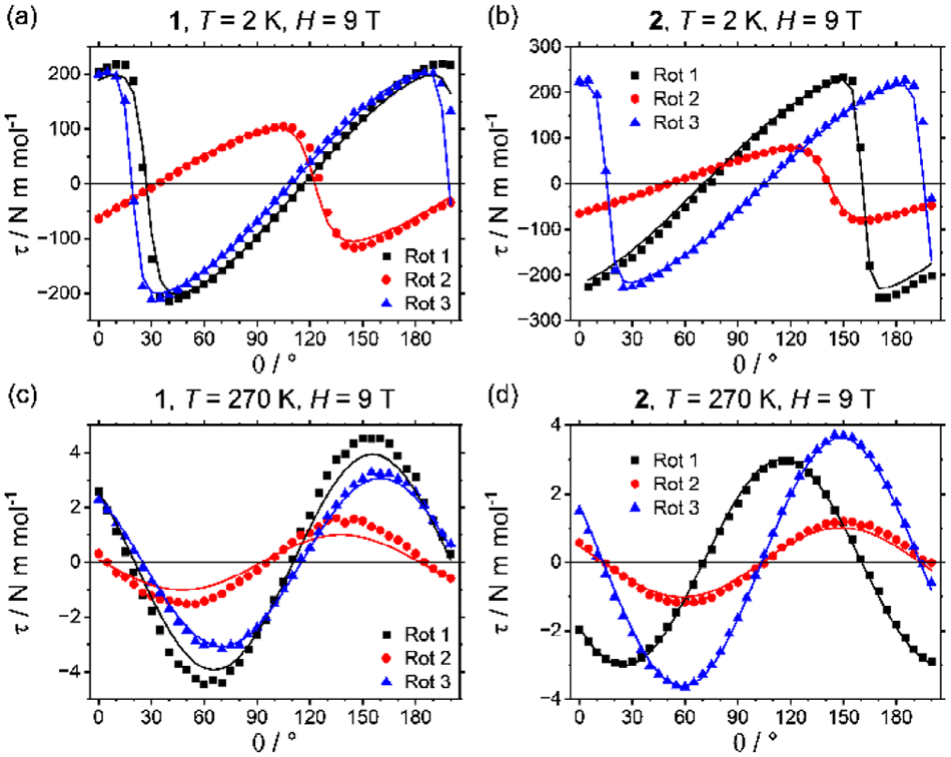
Torque at 2 K, 9 T for (a) **1** and (b) **2**, and at 270 K, 9 T for (c) **1** and (d) **2**, showing the three rotations. The solid lines indicate best‐fit curves. The black, red, and blue datapoints indicate the first, second and third rotations, respectively.

In **2**, at low temperature, *D*_xz_ is scanned vs *D*_y_ (Figure S13), giving an initial negative torque and an easy zero around 51–52° (2–10 K); at high temperature, *g*_y_ is scanned vs *g*_x_, giving an opposite phase of the initial torque and an easy zero at 106° (270 K) (Figure 4). The easy zero shifts with temperature as the easy direction swaps from being imposed by *D* to *g* anisotropy, a direct reflection of their noncollinearity (Figure 3). While in rotation 2 for **1** at low temperature, *D_z_* is scanned versus *D_xy_* (Figure S7), yielding an initial negative torque and an easy zero around 31–33° (2–50 K), where *D*_z_ is aligned with the field. At high temperature, *g*_yz_ is scanned vs *g*_x_, giving an opposite phase of the torque and an easy zero at 96° (270 K), where the average of *g*_z_ and *g*_y_ aligns with the field. The shift of the easy zero from *D*_z_ to *g*_yz_ is centerd at 150 K, where the torque signal goes through a minimum, this is reproduced in the simulation and suggests partial cancelation of the *D* and *g* anisotropy in this intermediate regime.

The model reveals that both compounds exhibit pronounced rhombic anisotropy, with zero‐field splitting parameters comparable to those reported for mononuclear Fe(II) complexes [6, 26]. The ZFS anisotropy is tending toward the easy axis, and the *g* anisotropy is tending toward the easy plane. The best fit results are reported in Table 1, together with ab initio results (see after) for comparison [27, 28] (Table 1).

### 2.2 DC Magnetic Susceptibility Measurement

**FIGURE 5:**
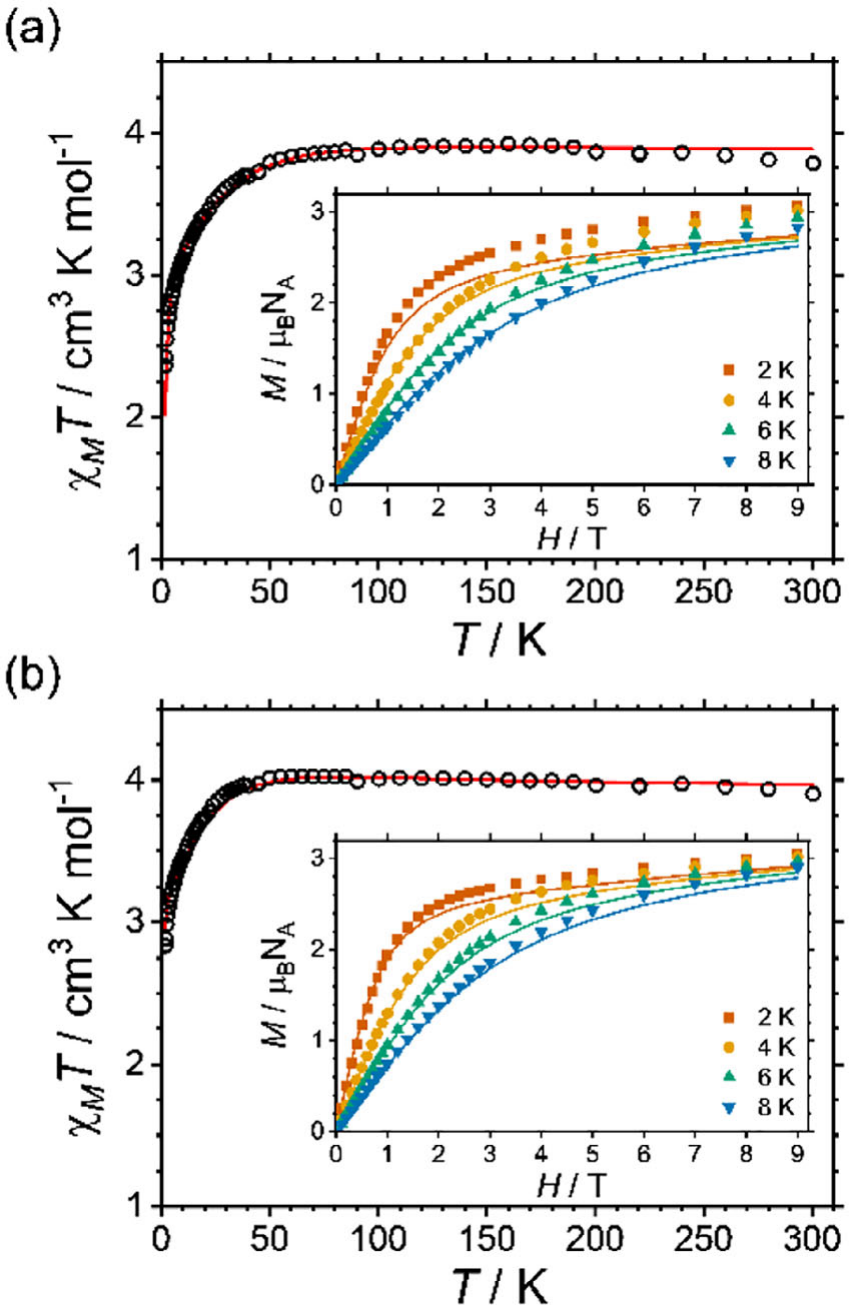
Temperature‐dependent magnetic susceptibility measurement performed on polycrystalline samples of (a) **1** and (b) **2** in the presence of 0.1 T external magnetic field. Open circles represent the experimental results, and solid lines represent the simulation using parameters derived from simultaneous fitting of experimental CTM and magnetic data. Inset: Field‐dependent magnetization data.

Temperature‐dependent magnetic susceptibility measurements on polycrystalline samples of **1** and **2** were performed between 2 and 300 K in the presence of a static magnetic field of 0.1 T. At 300 K, the observed *χ_M_T* values for **1** and **2** are 3.78 and 3.90 cm^3^ K mol^−^^1^, respectively, substantially higher than the value expected for a magnetically isolated high spin ion (*S* = 2, *g* = 2, *χ_M_T* = 3.0 cm^3^ K mol^−^^1^) (Figure 5) [27, 28]. This indicates that there is a non‐zero orbital contribution to the overall magnetic moment experimentally observed, which is not surprising considering the complex electronic structure with the first‐order orbital angular momentum. In both cases, the *χ_M_T* value remains nearly constant from room temperature down to 70 K before it precipitously drops below this temperature and reaches a final value of 2.36 and 2.83 cm^3^ K mol^−^^1^ at 2 K.

The overall observed *χ_M_T*(*T*) trend suggests that both complexes must possess significant zero field splitting, as expected from the best fitting values reported in Table 1. The field‐dependent magnetization measurements show a sharp increase without any clear saturation at *T* = 2 K and *B* = 9 T. The magnetic moment reaches a value of 2.85 and 3.01 *N_A_µ_B_* for **1** and **2,** respectively, both lower than the expected value of 4 *N_A_µ_B_* for an orbitally quenched *S* = 2. The non‐superimposable nature of the reduced magnetization data of both complexes further confirms the presence of magnetic anisotropy associated with the ground state of the complexes **1** and **2** (Figure S19). The EasySpin [25] program *curry* was incorporated into the customized script provided to *esfit* (see ESI for details) to optimize a single set of SH parameters over both CTM and static magnetic data. The experimental *𝜒_M_T(T)* data and *M(H)* data are well fit with a single set of SH parameters through the following formula, optimised over both CTM and static magnetic data.

(1) $${\hat{H}}_{s}=\mu_{B}\cdot \hat{S}\cdot g\cdot \vec{B}+D\left[{\hat{S}}_{z}^{2}-\frac{1}{3}S\left(S+1\right)\right]+E\left({\hat{S}}_{x}^{2}-{\hat{S}}_{y}^{2}\right)$$

Here, *D* and *E* are the axial and rhombic components of magnetic anisotropy tensor. The estimated *D* value (−25.6 cm^−1^ for **1** and −19.8 cm^−1^ for **2**) ranks among the largest reported for five‐coordinate Fe(II) complexes [11, 12, 29, 30]. Similar to the analogous Co(II) complexes, a smaller anisotropy is observed with heavier halides [13].

### 2.3 Theoretical Calculations

To elucidate the underlying cause of the negative zero‐field splitting observed in complexes **1** and **2**, we employed state‐averaged complete active space self‐consistent field (SA‐CASSCF) calculations, based on their x‐ray crystal structures. The active space was defined as CAS (6,5), encompassing 6 electrons within 5 orbitals, and included a comprehensive set of spin states, 5 quintets, 45 triplets, and 50 singlets. To incorporate dynamic electron correlation effects beyond the CASSCF level, we performed subsequent N‐electron valence perturbation theory (NEVPT2) calculations on the converged wavefunctions. This combined CASSCF/NEVPT2 methodology is well‐suited for accurately probing the electronic structure, zero‐field splitting parameters, and magnetic behavior of transition‐metal‐based systems.

**FIGURE 6:**
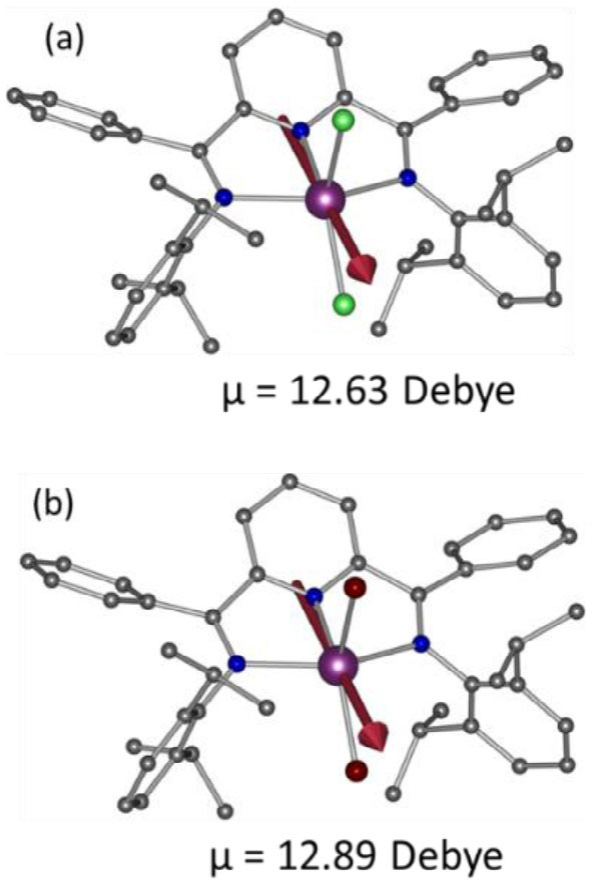
(a, b) The dipole moment vectors (maroon arrows) of **1** and **2,** respectively.

Prior to the dipole moment analysis, multireference CASSCF/NEVPT2 calculations were performed to accurately determine the magnetic anisotropy parameter (*D*). However, because the CASSCF treatment is restricted to the selected active space and does not explicitly account for the full contribution of ligand orbitals to the electron density distribution, it is not ideally suited for evaluating molecular electric dipole moments. Therefore, to obtain a more reliable description of the overall charge distribution and associated dipole moment, DFT calculations (B3LYP/LanL2DZ; see Computational Details) were carried out on the x‐ray geometry. The DFT‐computed dipole moment is presented in Figure 6. The computed dipole vector is not collinear with either halide bond; instead, it is tilted away from both the axial and basal halide directions, reflecting the combined influence of metal–ligand bonding asymmetry and ligand polarization effects. The degree of tilting can be quantified by the angles ∠X–Fe–*dip*, where X denotes the halide ligand, and *dip* represents the dipole moment direction. For complex **1**, the angles associated with the axial and basal halide bonds are 80.35° and 43.68°, respectively. In complex **2**, the corresponding angles are very similar, with values of 80.47° (axial) and 43.20° (basal). The consistently smaller angle relative to the basal halide indicates that the dipole moment is more closely aligned with the basal coordination environment, confirming that basal ligand polarization provides the dominant contribution to the overall dipole moment. The close similarity of these angles between the two complexes suggests that substitution of the halide ligand has a minor effect on the dipole moment orientation, while subtly modulating its magnitude through differences in electronegativity and polarizability. Overall, the dipole moment direction observed in the Figure 6 supports a picture in which intrinsic polarization arises primarily from basal ligand–metal interactions, in agreement with the computed electronic structure and experimental dielectric, PFM measurements.

To shed light on the sign and magnitude of the *D* value, we carried out CASSCF/NEVPT2 calculations, a methodology known to provide reliable numerical estimates of SH parameters in transition‐metal complexes [12, 13]. The computed Spin‐Hamiltonian (SH) parameters for complexes **1** and **2** are summarized in Table 1 and Table S6. For complexes **1** and **2**, the axial zero‐field splitting parameters (*D*) are calculated to be −29.87 and −29.78 cm^−^^1^, respectively, with corresponding rhombicity (*E/D*) of 0.316 and 0.232. Based on the computed multiplet splitting and the Δ/λ ≈ 1.6 criterion, the system lies outside the orbitally degenerate regime, justifying the applicability of the Spin Hamiltonian formalism (Table S7 and Figure S21). The computed *D* value for **1** shows good agreement with the experimental result, whereas that for **2** is slightly overestimated. The calculated *g*‐tensor components for **1** are *g_x_* = 1.902, *gᵧ* = 2.247, and *g_z_* = 2.652, while for **2** they are *g_x_* = 1.925, *gᵧ* = 2.120, and *g_z_* = 2.628. These values indicate pronounced magnetic anisotropy and are in good agreement with the experimentally determined SH parameters (Table 1), despite a slight overestimation of the rhombicity (|*E/D*|). The computed magnetic susceptibility and magnetization also aligned with the experimental observations (Figures S22 and S23). The orientation of the magnetic anisotropy axes (*D_z_* and *g_z_*) is nearly colinear, with deviation angles of 3.626° for **1** and 4.485° for **2**, in good agreement with the CTM data for **2** (CTM: 8.4°) and overestimated alignment for **1** (CTM: 22.5°). The orientations of the intermediate and hard *g* and *D* anisotropy axes are poorly reproduced by NEVPT2 (Figures S15–S18, S20). Notably, the principal magnetic anisotropy axes align along one of the X^−^ ligands situated in the equatorial plane of the square‐pyramidal geometry (Figures S15 and S17).

**FIGURE 7:**
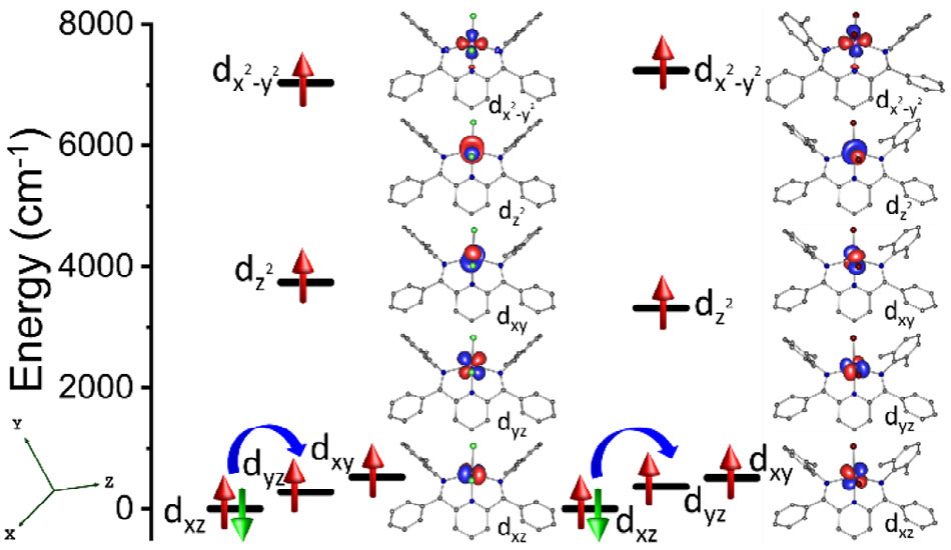
NEVPT2‐AILFT d‐orbital energy level diagram for complexes **1** and **2** with the d‐orbitals using isosurface value 0.0334.

Analysis of the NEVPT2‐computed excited states reveals that the first two excited states predominantly contribute to the overall zero‐field splitting of both complexes. State‐wise decomposition analyzes show that the first excited state contributes negatively to *D*, whereas the second excited state contributes positively (Figure 7 and Tables S7, S8). Because the magnitude of the first excited‐state contribution is larger, both complexes exhibit an overall negative *D* value, stabilizing axial magnetic anisotropy, fully consistent with CTM measurements. The ground‐state wavefunction analysis indicates strong multiconfigurational character in both complexes (Tables S8 and S9), reflecting significant d‐orbital mixing induced by the reduced molecular symmetry and resulting in deviations from ideal trigonal‐bipyramidal or square‐pyramidal splitting patterns. In both **1** and **2**, the dominant negative contribution to *D* arises from the lowest‐energy, spin‐conserved transition between orbitals of the same |m_l_| value (d_xz_ → d_yz_), whereas the higher‐energy transition between orbitals of different |m_l_| values (d_xz_ → d_xy_) contributes positively to *D*, consistent with established theoretical frameworks [31].

To probe the nature of metal–ligand bonding in **1** and **2**, ab initio ligand field theory (AILFT) calculations were performed (Table S10). Both spin–orbit coupling (ζ) and Racah parameters (B, C) reveal notable nephelauxetic effects compared to the free‐ion values. The ζ values decrease to 398.1 cm^−1^ (**1**) and 389.6 cm^−1^ (**2**), corresponding to 4.23% and 6.27% reductions, while B and C are reduced by ∼9.4% and ∼8.2%, respectively, consistent with significant Fe–ligand covalency. The slightly larger reduction for **2** reflects its softer donor character, enhancing orbital mixing and modulating spin–orbit coupling. The nearly constant C/B ratios (∼3.95 vs. 3.90 for the free ion) indicate proportional reductions in B and C. Overall, these results confirm pronounced metal–ligand covalency in both complexes, stronger in **2**, which directly impacts zero‐field splitting and magnetic anisotropy.

### 2.4 Magneto‐Structural Correlation

To disentangle the relative contributions of electronic and steric effects on the magnetic anisotropy of Fe(II) centers, we carried out systematic ligand‐field modeling based on the structural framework of **1**. For probing electronic effects, the halide ligand was systematically varied (–─F, –─Cl, –─Br, –─I) alongside pseudohalides (–‐NCS, –‐SCN) mentioned as **1‐F,** **1** **‐I**, **1‐NCS**, **1‐SCN** while maintaining the same ligand coordination environment (Table S11). To evaluate steric influences on the model complexes and the parent complexes, we introduced an additional methyl substituent on the ligand backbone **1‐F (^t^Bu)**, **1‐Cl (^t^Bu)**, **1‐Br (^t^Bu), 1‐I (^t^Bu), 1‐NCS (^t^Bu)**, and **1‐SCN (^t^Bu)**, thereby increasing steric bulk without altering the donor atom identity. These calculations reveal that the combination of a soft halide donor (–‐I) with a sterically encumbered ^t^Bu‐substituted ligand produces the most favorable magnetic anisotropy. This outcome can be attributed to the synergistic effect of strong spin–orbit coupling from the heavier halide and the steric hindrance imposed by the bulky substituent, which together enhance the out‐of‐plane displacement of the Fe(II) ion and maximize axial anisotropy. Notably, this trend parallels that observed in Co(II)–NNN pincer complexes, where an increased out‐of‐plane shift has likewise been shown to reinforce axial anisotropy reported by us and others [13, 23]. Thus, our design strategy not only disentangles the role of electronic versus steric contributions but also highlights a common structural principle across Fe‐ and Co‐based NNN pincer complexes, providing a rational pathway for tailoring anisotropy in this family of molecular systems.

### 2.5 AC Magnetic Data

Given that both complexes possess an easy‐axis magnetic anisotropy, temperature and frequency‐dependent AC magnetic susceptibility measurements were performed on polycrystalline samples to elucidate their magnetic relaxation behavior. In zero applied magnetic field, no out‐of‐phase (*χ*_*M*_ *″*) signals were detected for either of the complexes, indicating that ground‐state quantum tunnelling of the magnetization (QTM) dominates the relaxation process (Figures S24 and S25). This observation is fully consistent with the pronounced rhombicity extracted from the CTM analysis for complexes **1** and **2**. Field‐sweep AC measurements were performed to identify the optimum external magnetic where the magnetization relaxation is slow. Even at the optimal bias field (4.8 kOe), the frequency‐dependent AC measurements show only a very weak *χ_M_″* signal, indicating that the applied external magnetic field is not sufficient enough to quench/suppress the QTM completely. Such field‐dependent relaxation behavior, characterized by the absence or extreme attenuation of zero‐field *χ_M_″* signals, has likewise been reported in several iron‐based single‐ion magnets, underscoring the sensitivity of Fe(II) systems to QTM when significant rhombicity is present [32, 33].

### 2.6 Ferroelectric Studies

**FIGURE 8:**
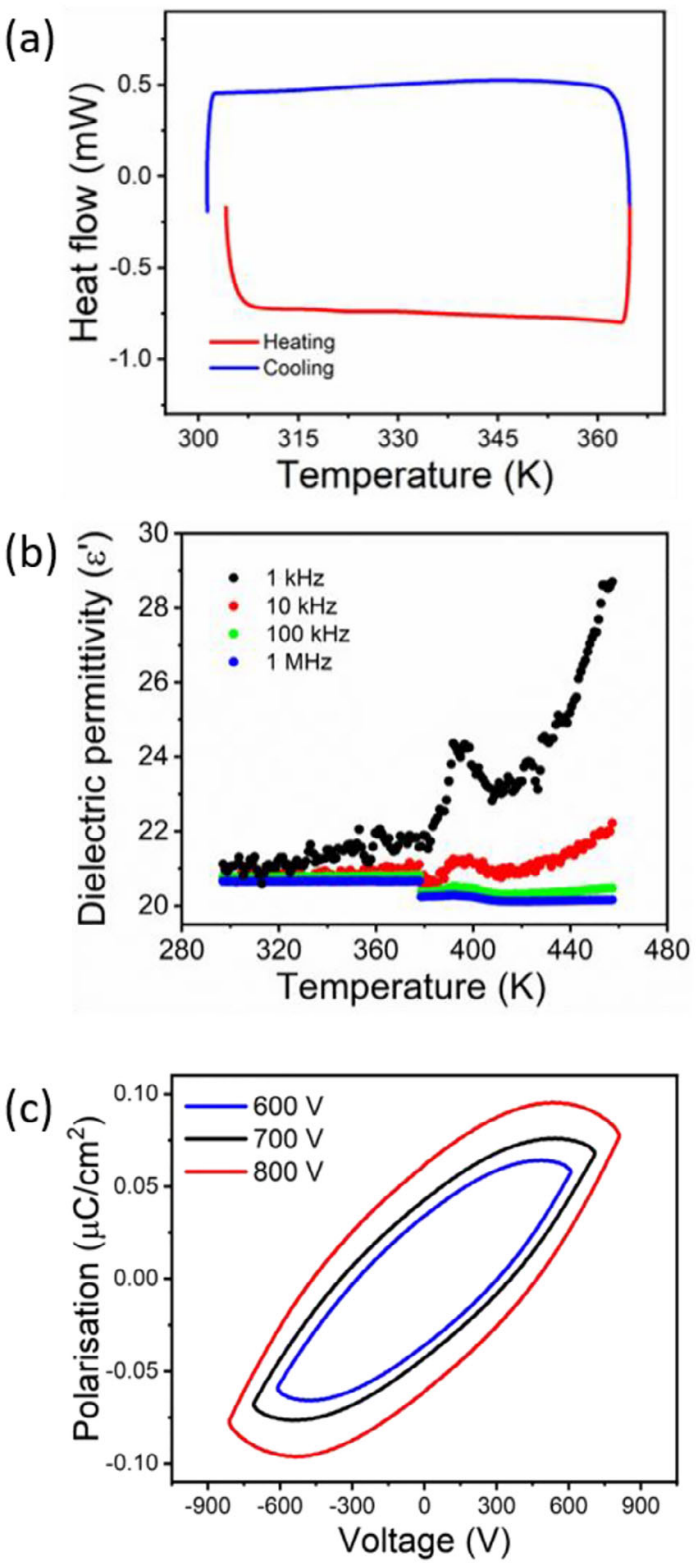
(a) DSC curves for heating and cooling runs (b) Dielectric constant (ɛ’) vs Temperature (d) P‐E hysteresis loop of **2**.

Complexes **1** and **2** crystallize in the polar, non‐centrosymmetric space group *P1*, which is well‐suited for ferroelectric applications. Thermogravimetric analysis (TGA) reveals weight losses of 14% for **1** and 12.6% for **2**, commencing at 360 K, consistent with the release of the chloroform molecule present in the unit cell (Figures S26a and S27a). Differential scanning calorimetry (DSC) measurements up to 360 K show no thermal anomalies, indicating the absence of any structural phase transitions within this temperature range (Figure 8b and Figure S26). Powder X‐ray diffraction (PXRD) profiles demonstrate that the complexes retain the polar ferroelectric phase below 353 K, while distinctly different diffraction patterns are observed above this temperature (Figure S27b). Accordingly, the Curie temperature (*T*_c_; temperature at which ferroelectric to paraelectric transition occurs) of the complexes is estimated to be around 350 K, which is significantly above room temperature and is both rare and challenging to achieve in molecular materials [34, 35].

Consistent with this behavior, the dielectric permittivity (ε′), measured on pressed pellets under an applied AC electric field, remains nearly constant up to approximately 353 K. Beyond this temperature, a pronounced dielectric anomaly appears, due to loss of chloroform solvent present in the unit cell with peak ε′ values of 10.73 and 24 (at 1 kHz) at 400 K for complexes **1** and **2**, respectively, accompanied by an increase in dielectric loss (tan δ) (Figure 8b and Figures S26,S27). The continued increase in dielectric permittivity around 500 K is attributed to the onset of melting of the complexes. Attempts to obtain single‐crystal X‐ray diffraction data at elevated temperatures were unsuccessful, as the crystals lost crystallinity upon further heating; a definitive structural origin for the anomalous dielectric features observed around 450 K could not be established.

The ferroelectric characteristics of **1** and **2** were substantiated through polarization–electric field (P–E) hysteresis measurements conducted on pressed pellets at room temperature at an operating frequency of 1 Hz, yielding spontaneous polarization (P_s_) values of 0.03 and 0.08 µC cm^−^^2^, respectively (Figure 8c and Figure S28a). The observed lossy character of the hysteresis loops arises from the intrinsic (semi)conducting behavior of metal complexes, which is been well documented by us and others elsewhere [36, 37, 38, 39, 40, 41, 42]. Furthermore, positive‐up negative‐down (PUND) measurements performed on **2** (Figure S28b) corroborate the switchable polarization and confirm its ferroelectric response.

To probe the local piezoelectric properties of the complexes, we conducted piezo response force microscopy (PFM) on a single crystal at room temperature (Figure S30). The vertical and lateral PFM (vector PFM) images clearly reveal the orientation of different domains in both complexes.

**FIGURE 9:**
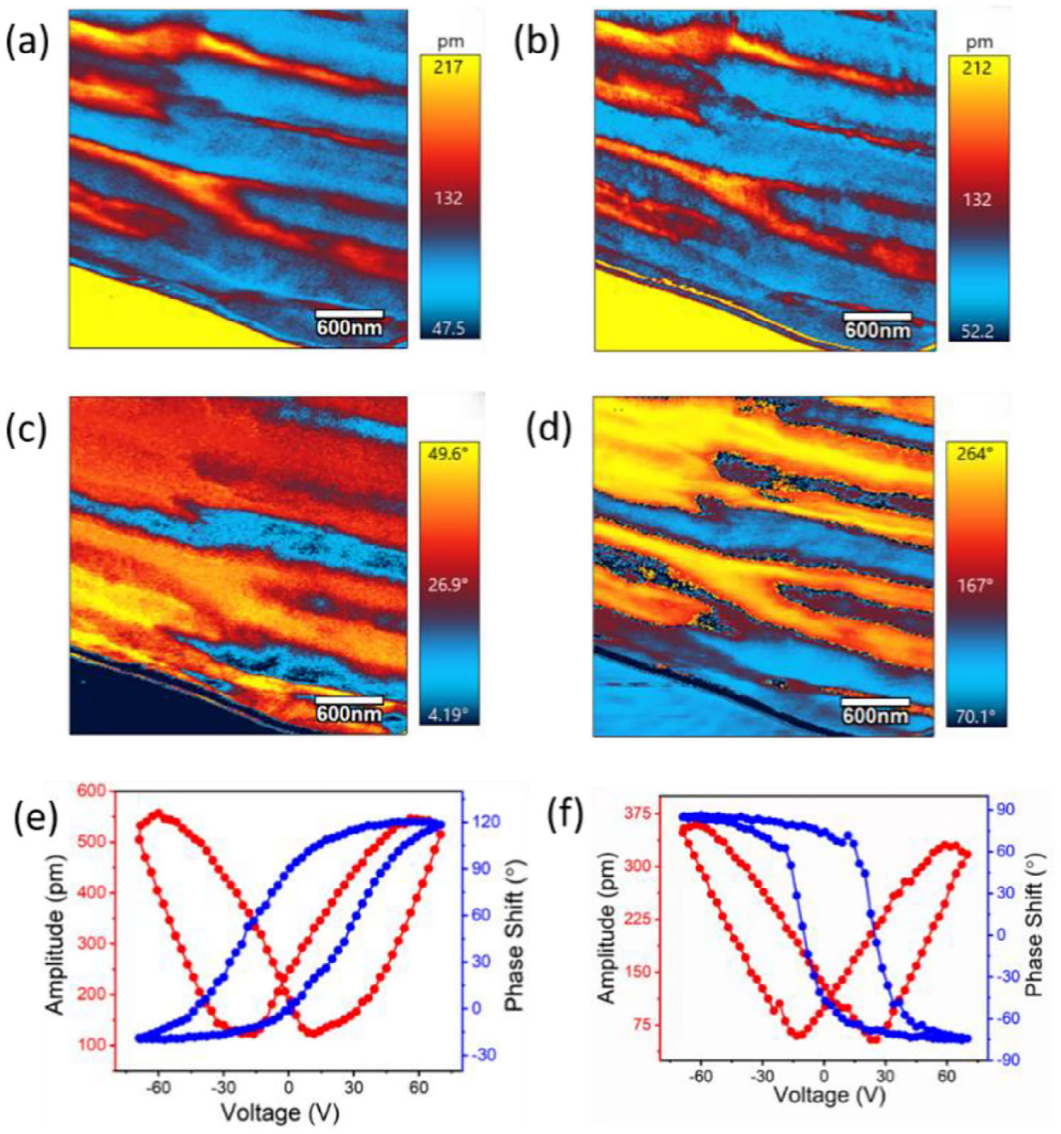
(a, b) Vertical PFM amplitude and phase (c, d) Lateral PFM amplitude and phase showing the different domains of **2** measured on a single crystal (e, f) OFF‐state amplitude‐Voltage butterfly loop and Phase Shift‐Voltage hysteresis loop of **2** and **1,** respectively.

The surface topography exhibits features clearly distinct from those observed in the PFM images, confirming that the measured signals originate from polarization rather than from variations in surface morphology (Figure 9a–d and Figure S29–S31). Switching spectroscopy PFM (SS‐PFM) measurements yielded an OFF‐state butterfly‐shaped amplitude–voltage curve, and phase–voltage hysteresis loop with a 180° phase shift, showing the polarization switching induced by the electric field (Figure 9e,f). The magnitude of the piezo‐electric coefficient (d_33_) value is found to be 5 ± 0.5 and 11 ± 0.5 pm V^−1^, from the OFF‐state graph, which was calculated using the equation, d_33_ = ∆z/V (where ∆z is the deflection of the cantilever caused by the deformation of piezoelectric samples under applied electric field and V is the applied voltage due to converse piezoelectric effect). Under varying tip DC voltages (−20 to +20 V), the phase and amplitude loops of **2** display bias‐dependent asymmetry, which illustrates typical ferroelectric behavior. (Figure S32). The OFF‐state amplitude and phase curves of **1** & **2** measured over multiple cycles exhibit reproducible, non‐drifting loops, confirming the absence of transient charge‐accumulation effects (Figure S33) [43].

Since the complexes are truly isostructural, the observed differences arise solely from halide substitution. Replacement of Cl (**1**) with Br (**2**) introduces a heavier, more polarizable halide ion, which enhances lattice polarizability and softens the metal–halide bonding environment [44]. This enhanced polarizability arises from greater electric‐field‐induced lattice distortions, analogous to the mechanism responsible for the increased spin–electric coupling in five‐coordinate complexes [20, 22, 45]. These effects rationalize the significantly higher d_33_ value of **2** [44, 46, 47, 48]. This approach underscores how subtle chemical modification can be leveraged to optimize material properties for functional applications.

## 3 Conclusion

We have synthesized and structurally characterized two five‐coordinate, distorted square‐pyramidal Fe(II) complexes, [Fe(L)(X)_2_]·CHCl_3_ (X = Cl (**1**), Br (**2**)), crystallizing in a non‐centrosymmetric polar space group. Using oriented‐crystal CTM measurements, we have accurately determined the sign, magnitude and orientation of the both the ZFS and *g* tensor, with *D* = −25.6(4) cm^−^^1^ (*E*/*D* = 0.208(8)) for **1** and *D* = −19.8 cm^−^^1^ (*E*/*D* = 0.144(5)) for **2** representing, to the best of our knowledge, the first reliably quantified electronic structure and spin‐Hamiltonian parameters for a mononuclear Fe(II) system using this methodology. The ZFS easy axis is aligned along the equatorial iron‐halide bond. Experimentally, we observe different orientations of the *D* and *g* anisotropy tensors. While *g*_z_ is near collinear with the ZFS easy axis in **2**, it is tilted by 22.5° in **1**. The intermediate axes and hard axes differ significantly between the *g* tensor and *D* tensor in both **1** and **2**. A single set of parameters can be used to describe the CTM and the experimental magnetic data (*χ_M_T(T)* and *M(H)*), which further validates the robustness of our analysis. Notably, the large negative *D* values rank among the highest reported for Fe(II) in this coordination geometry. Structural subtleties, including halide substitution and out‐of‐plane Fe(II) displacement, are shown, via ab initio CASSCF/NEVPT2 calculations, to play a decisive role in governing the magnetic anisotropy. Complementary dielectric, thermal, PFM, and P‐E loop investigations confirm intrinsic polarization, with complex **2** exhibiting a slightly higher piezoelectric response, consistent with computed dipole moments that suggest enhanced lattice deformation under applied electric fields. Together, these results constitute the first systematic exploration of coexisting magnetic and electric functionalities in an Fe(II) molecular system, offering a clear design blueprint for intrinsic spin–electric coupling. Overall, this work broadens the landscape of Fe(II)‐based single‐ion magnets and establishes a foundational platform for the development of multifunctional molecular materials for next‐generation spin–electric devices and quantum technologies.

## Conflicts of Interest

The authors declare no conflicts of interest

## Supporting information




**Supporting File**: advs75759‐sup‐0001‐SuppMat.pdf.

## Data Availability

The data supporting this article have been included as part of the ESI.† The crystallographic data have been deposited with the CCDC under the accession numbers 2524289 (**1**) and 2524290 (**2**).
