# Supplementary material for: Quantifying Magnetic Anisotropy of Ferroelectric Fe(II) Square‐Pyramidal Systems Using Torque Magnetometry
Source: Adv Sci (Weinh). 2026 May 20:e75759. Online ahead of print. doi: 10.1002/advs.75759 (PMC13336093; doi:10.1002/advs.75759)
Supplement: Supplementary file 1 — Supporting File: advs75759‐sup‐0001‐SuppMat.pdf. [file ADVS-9999-e75759-s001.pdf]

Quantifying Magnetic Anisotropy of Ferroelectric Fe(II) Square-Pyramidal Systems Using Torque Magnetometry

Vijaya Thangaraj, [a] Gemma K. Gransbury, [b] Deepanshu Chauhan, [a] Dipanti Borah [a], Gopalan Rajaraman, *[a] Mauro Perfetti, *[b] Maheswaran Shanmugam*[a]

[a] Department of Chemistry,

Indian Institute of Technology Bombay,

Powai, Mumbai 400076, Maharashtra (India)

E-mail: rajaraman@chem.iitb.ac.in (GR); eswar@chem.iitb.ac.in (MS)

[b] Dipartimento di Chimica "Ugo Schiff" and UdR INSTM

Università degli Studi di Firenze, Via della Lastruccia 3−13, 50019 Sesto Fiorentino, Italy.

E-mail: mauro.perfetti@unifi.it

Materials and Methods

All reactions were carried out under an inert Argon atmosphere. Chemicals and solvents were purchased from commercially available sources (Sigma Aldrich/Thermofisher Scientific). They were used without further purification, except for aniline, which was freshly distilled before being used for ligand synthesis. Toluene was freshly dried before use. The [2,6-bis{1-[(2,6-diisopropylphenyl)- imino]benzyl}pyridine]) ligand (L) was synthesized as per the literature report. [1]

Single crystal X-ray diffraction measurements: Single crystal X-ray diffraction measurements were performed on a Bruker diffractometer with MoKα radiation (λ = 0.71073 Å). Cell refinement and data reduction were performed using CrysAlisPro 1.171.38.43. The Crystal data was solved by direct methods and then refined by full-matrix least-squares refinements based on F^2 using the SHELXL, as implemented in Olex2-1.5. All non-hydrogen atoms were refined anisotropically, and all hydrogen atom's positions were generated geometrically. (CCDC Number: 2524289-2524290)

Powder X-ray diffraction measurement (PXRD): Powder X-ray diffraction (PXRD) was performed on a RigakuD/tex Ultra 250 instrument using a Cu Kα . The diffraction patterns were collected in the 2θ range of 5°−50° with a step size of 0.01.

Elemental analysis: Elemental analysis was performed on an Elementar Vario MICRO cube microanalyzer.

Magnetic measurement: Magnetic susceptibility measurements were carried out on a Quantum Design SQUID magnetometer MPMS-3, operated between 1.8 to 300 K for DC-applied fields ranging from 0 to 70 kOe. Alternating current (AC) susceptibilities were carried out under an oscillating AC field with frequencies ranging from 0.1 Hz to 1 kHz.

Cantilever Torque Measurement (CTM): Torque measurements were performed on a Quantum Design PPMS using the torque insert and standard 2-leg piezoelectric torque chip. The empty torque chip was first calibrated for a broad range of temperatures and magnetic fields, encompassing the experimental ranges. The sample was measured in temperatures of 2-270 K and magnetic fields of 0-9 T (0 T for further calibration). The signal was kept to < 10^{-5} N m, within the linear response regime of the cantilever, as reported by the manufacturer. During each measurement, the crystal was rotated from 0–200° around an axis perpendicular to the external magnetic field in 5° increments (+θ corresponds to sample rotating clockwise). The rotation axes for each of the three rotations and direction of the magnetic field at zero angle in the *ab'c** reference frame commonly used in cantilever torque magnetometry are given in Table S4. Images of the initial molecule orientations are shown in Figure S8 (**1**) and S9 (**2**). The single crystal of **1**

was a dark green rod, weighing 6 μg and the single crystal of **2** was a green prism, weighing 16 μg; in the fitting procedure these masses were revised to 4.61(2) μg for **1** and 11.37(4) μg for **2**. Each crystal was secured on an acetate plate with N-grease and crystallographically indexed. For the Single-crystal X-ray indexing, an Oxford Diffraction Xcalibur3CCD four-circle diffractometer with a graphite monochromator and Mo-Ka radiation was used.

The zero field dataset at each temperature was fit to a quartic polynomial using *polyfit* in MATLAB and subtracted from the in-field signal, to account for gravitational effects. The exception was 200 K as the subtraction made the torque curve aperiodic. A shift in the baseline of the torque signal was observed with field, to correct for this the average value (25–200°) was subtracted from each curve. The torque curves and static magnetic data were simultaneously fit using a customized function in the EasySpin program *esfit* using the Nedler-Mead simplex algorithm, starting from a good simulation. The fitting parameters were rhombic g values [g_x, g_y, g_z], ZFS values D and E/D where the diagonal elements of the D-tensor [D_x, D_y, D_z] are equal to [$-D/3 + E$, $-D/3 - E$, $2D/3$], the orientations of the g- and D-tensors in the molecular frame (ab′c*) given by Euler angles (α_g, β_g, γ_g) and (α_D, β_D, γ_D), respectively (active intrinsic rotations, z-y′-z″), and a scaling parameter to account for uncertainty in the single crystal mass, effectively scaling the absolute values of the torque. The torque was calculated as the negative partial derivative of the free energy with respect to θ in radians, calculated using the finite difference method with $\Delta\theta = 0.001°$. The magnetic data were calculated using the EasySpin function *curry* with the susceptibility equal to M/H. A weighting scheme was devised to give an approximately equal contribution of all temperatures and rotations of the torque, and appropriate contribution of static magnetic data to the error. The experimental and simulated torque data at each temperature was divided by the maximum experimental torque measured at 9 T. All curves for a particular rotation and temperature (experimental and simulated) were further weighted by $25/\sum H$ where $\sum H$ is the sum of the fields (in T) under those conditions, to provide approximately equal weights of each temperature regardless of the number of fields measured. To account for the error in the measured mass (due to the precision and accuracy of the balance and contribution of oil on the crystal), the simulated torque was scaled using the fitted variable mentioned above. The experimental and simulated isothermal magnetisation data were divided by the maximum value of the magnetization at 2 K, 9 T. The minimum and maximum experimental values of χT were used to normalise the experimental and simulated χT (0 to 2). The fitted curves provide excellent agreement with experiment (Figure S10-S15).

The fitted g-tensor Euler angles were (83.5(13), 77.3(4), 6.3(7))° for **1** and (98.0(2), 59.8(3), 86.8(4))° for **2**. The fitted D-tensor Euler angles were (99.6(3), 60.5(3), 69.8(14))° for **1** and (88.4(3), 58.53(12), 44.5(10))° for **2**. This gives rise to g and D tensors in the ab′c* reference frame given in Table S5, visualised in Figure S16 (**1**) and S17 (**2**).

Dielectric Measurement: Complex dielectric permittivity was measured with Keysight Impedance Analyzer E4990A system, where two parallel plate capacitor geometry is considered. Silver conductive paste deposited on both sides of the pressed pellets of the sample were used as top and bottom electrodes.

Piezoresponse Force Microscopy (PFM) measurements: Polarization domain imaging, piezoelectric properties and ferroelectric switching were studied on pressed pellet by scanning probe microscopy (SPM) technique using piezo-response force microscopy (PFM) mode in a MFP-3D BIO instrument (Asylum Research) using an SCM-PIT-V2 probe. Local topography and piezoelectric properties were investigated in Dual AC Resonance Tracking (DART) mode, and the phase and amplitude responses were further quantified using Switching Spectroscopy PFM (SS-PFM). For the present study, the writing voltage was varied between −20 V and +20V, with a hold time of 50 ms for each segment. All phase and amplitude signals were extracted exclusively from the off-field segments, ensuring that the response originates from genuine remnant polarization rather than capacitive or electrostatic artifacts.

P-E Loop Measurement: Ferroelectric and PUND measurements were carried out at room temperature on the pressed pellets, utilizing a Radiant Precision II ferroelectric loop tracer equipped with a high-voltage amplifier from Radiant Technologies USA.

Computational Details: We performed *ab initio* single-point calculations using the ORCA 5.0 program suite to investigate the electronic structure and magnetic properties of the Fe(II) centre, focusing on the extraction of zero-field splitting (ZFS) parameters, g-tensors, and low-lying electronic states. The molecular coordinates were taken directly from the crystallographic data, with no further geometry optimization. Scalar relativistic effects were addressed using the Douglas–Kroll–Hess (DKH) formalism. For the basis sets, we applied the DKH-adapted versions: def2-TZVP for Fe, def2-TZVP(-f) for Cl and N atoms, and def2-SVP for all other atoms. The orbital optimization was carried out via the state-averaged complete active space self-consistent field (SA-CASSCF) method, using a CAS(6,5) active space that incorporates 6 electrons in 5 orbitals. Within this framework, all 5 quintets, 45 triplets, and 50 singlets were evaluated. To account for dynamic correlation effects, we subsequently performed NEVPT2 (N-electron valence second-order perturbation theory) calculations on the converged CASSCF wavefunction. Furthermore, ab initio ligand field theory (AILFT) was utilized to derive accurate d-orbital energies for the Fe(II) complex. **Geometry optimisation**: We employed the Gaussian16 suite for the geometry optimization of all model complexes. Specifically, the optimizations were performed using the unrestricted B3LYP functional, with the LanL2DZ basis set applied to the Fe(II) ion and the 6-31G* basis set for the remaining atoms. This combination has proven to be a reliable and widely adopted methodology for Fe(II) complexes.

Synthetic procedure for [Fe(L)Cl_2] (1)

Anhydrous $FeCl_2$ (X = Cl or Br, 1 mmol) was added to a clear yellow solution of the NNN-pincer ligand (L, 1 mmol) in dry toluene (8 mL). The reaction mixture was refluxed for 12 h, during which the solution gradually turned dark green and a solid residue formed. The resulting solid was collected by filtration and washed with hexane. Block-shaped single crystals suitable for X-ray diffraction were obtained the following day by dissolving the residue in chloroform and carefully layering with hexane. Yield of **1** = 30 % (Based on ligand). Elemental analysis details: Calc. (%) C, 62.03; H, 5.68; N, 4.93. Found (%) C, 61.95; H, 5.030; N, 4.51.

Synthetic procedure for [Fe(L)Br_2] (2)

A similar synthetic procedure was followed as in **1** to isolate **2**, but anhydrous $FeBr_2$ was used in place of $FeCl_2$. Moreover, the reaction mixture was stirred at room temperature for overnight (unlike in the case of **1**). Yield of **2** is 35 % (Based on ligand). Elemental analysis details: Calc. (%), 56.17; H, 5.14, N, 4.46. Found (%) C, 56.16, H, 4.48, N, 4.17.

Table S1: Crystallographic parameters of **1-2.**

	**1**	**2**
Empirical formula	$C_{44}H_{48}Cl_5FeN_3$	$C_{44}H_{48}Br_2Cl_3FeN_3$
Formula weight	851.95	940.87
Temperature/K	150.00(10)	150.00(10)
Crystal system	triclinic	triclinic
Space group	*P1*	*P1*
a/Å	9.6210(3)	9.5893(3)
b/Å	9.6598(2)	9.8311(2)
c/Å	13.7332(3)	13.7163(4)
α/°	93.350(2)	94.099(2)
β/°	107.666(2)	107.251(3)
γ/°	114.309(2)	114.035(2)
Volume/Å^3	1083.32(5)	1100.10(6)
Z	1	1
ρ_{calc}g/cm^3	1.306	1.420
μ/mm^{-1}	0.690	2.374
F(000)	444.0	480.0
Crystal size/mm^3	0.32 × 0.25 × 0.2	0.1 × 0.25 × 0.15
Radiation	Mo Kα (λ = 0.71073)	Mo Kα (λ = 0.71073)
2Θ range for data collection/°	3.184 to 49.994	3.188 to 50
Index ranges	$-11 \leq h \leq 11$, $-11 \leq k \leq 11$, $-16 \leq l \leq 16$	$-11 \leq h \leq 11$, $-11 \leq k \leq 11$, $-16 \leq l \leq 16$
Reflections collected	40065	41083
Independent reflections	7666 [R_{int} = 0.0788, R_{sigma} = 0.0625]	7772 [R_{int} = 0.0602, R_{sigma} = 0.0412]
Data/restraints/parameters	7666/3/486	7772/3/486
Goodness-of-fit on F^2	1.042	1.031
Final R indexes [I>=2σ (I)]	R_1 = 0.0492, wR_2 = 0.1098	R_1 = 0.0344, wR_2 = 0.0758
Final R indexes [all data]	R_1 = 0.0599, wR_2 = 0.1177	R_1 = 0.0371, wR_2 = 0.0777

Table S2: Summary of SHAPE analysis for **1** and **2**.

Complexes	PP-5	vOC-5	TBPY-5	SPY-5	JTBPY-5
**1**	32.962	4.402	5.692	**2.450**	8.710
**2**	32.737	4.997	6.057	**2.945**	9.707

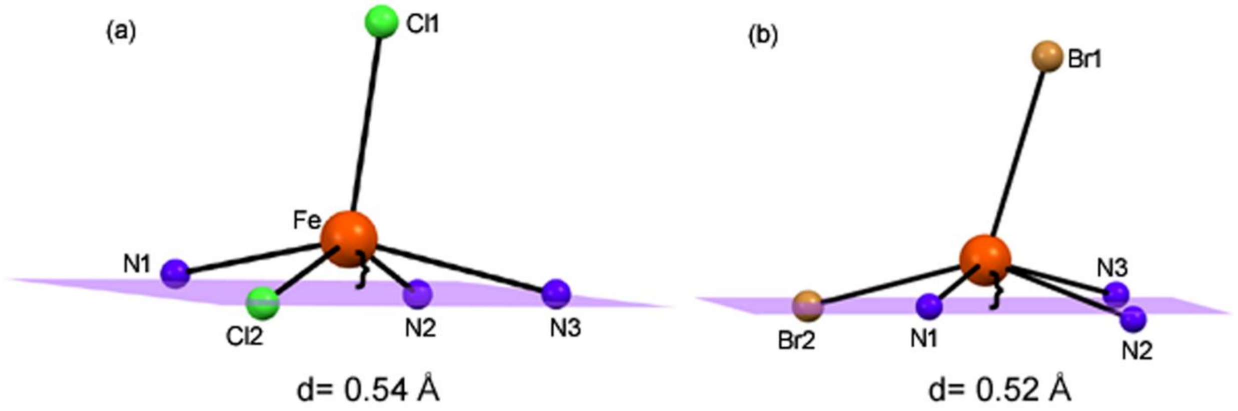

Figure S1: (a,b) Out-of-plane shift of Fe(II) metal center from the square basal plane for **1** and **2**.

Table S3: Selected bond lengths and bond angles of **1** and **2.**

**Bond angle (°)**	**1**	**2**
X1- Fe1-X2	124.03(7)	123.64(16)
N1- Fe1-X2	97.16(13)	100.93(7)
N1- Fe1-X1	97.44(13)	97.77(7)
N2- Fe1-X2	89.32(14)	88.18 (7)
N2- Fe1-X1	146.56(15)	148.07(7)
N3- Fe1-X2	98.05(13)	98.18(7)
N3- Fe1-X1	100.56(13)	95.66(7)
**Bond length(Å)**	**1**	**2**
Fe1-X2	2.351(17)	2.38(4)
Fe1-X1	2.246(16)	2.50(4)
Fe1-N1	2.231(5)	2.23(3)
Fe1-N2	2.062(5)	2.06(3)
Fe1-N3	2.239(5)	2.22(3)

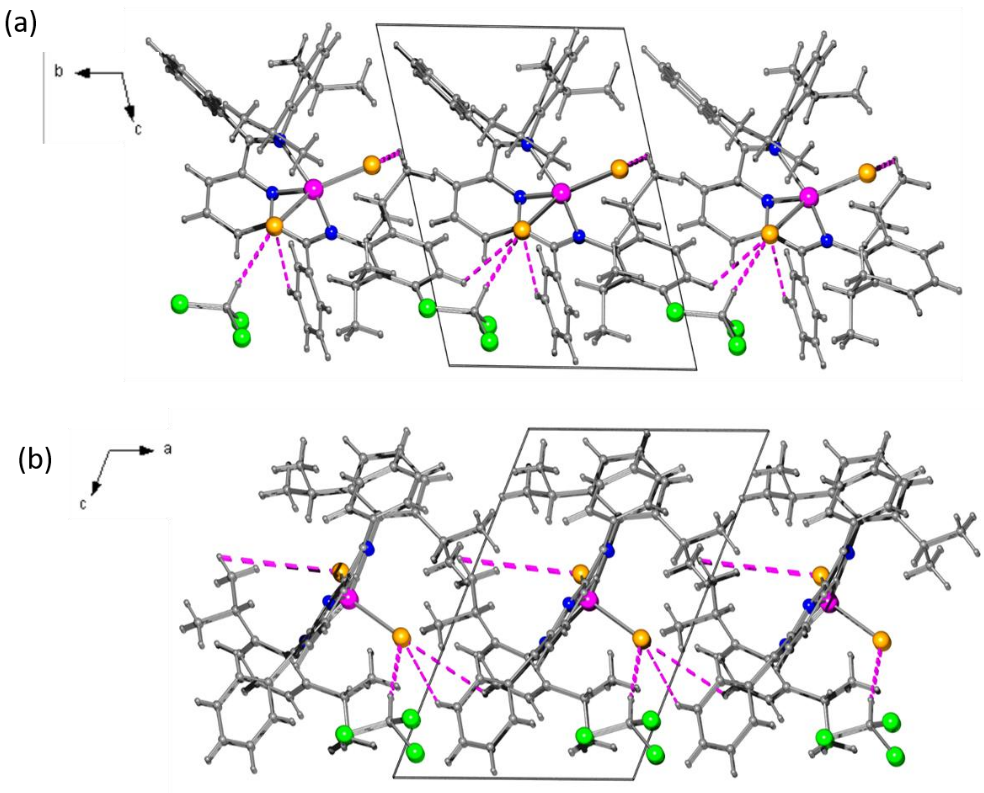

Figure S2: Packing diagram of **2** along the a-axis (b) along the b-axis. Magenta dotted lines represent intermolecular hydrogen bonding

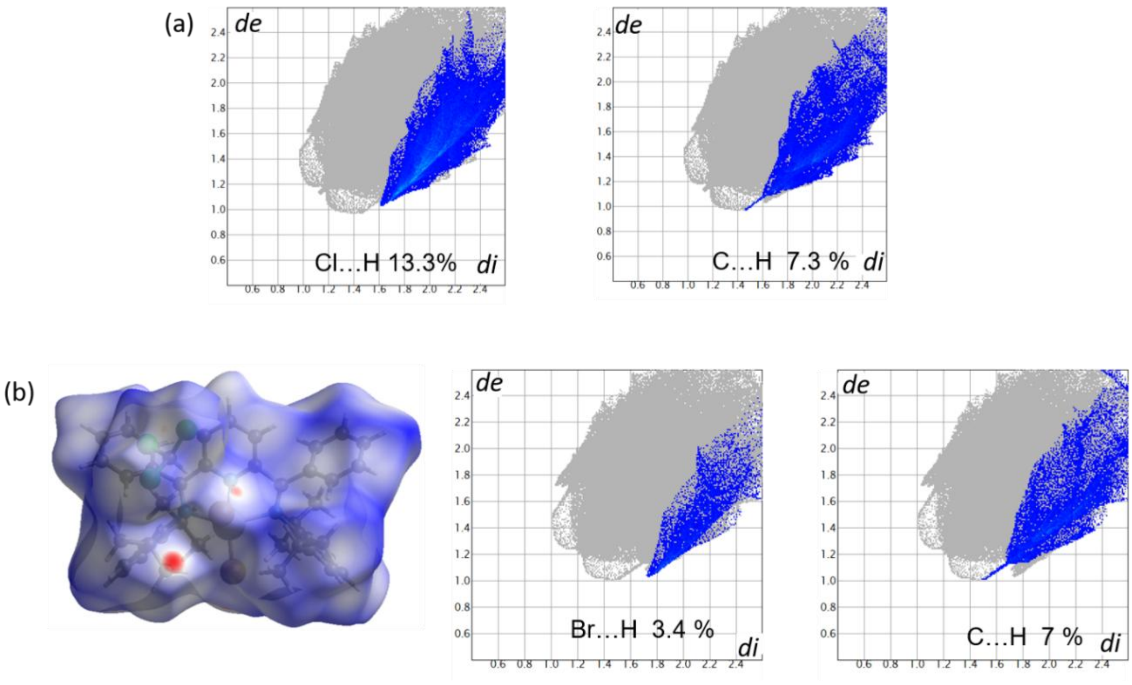

Figure S3: (a) Hirshfeld two-dimensional fingerprint plots of **1** showing Cl···H (13.3 %) and C···H (7.3 %) interactions (b) Hirshfeld surface view of **2** and two-dimensional fingerprint plots of Br···H (3.4 %) and C···H (7 %) interactions. Here *di* and *de* denote the distances (Å) to the nearest atoms inside and outside the surface, respectively.

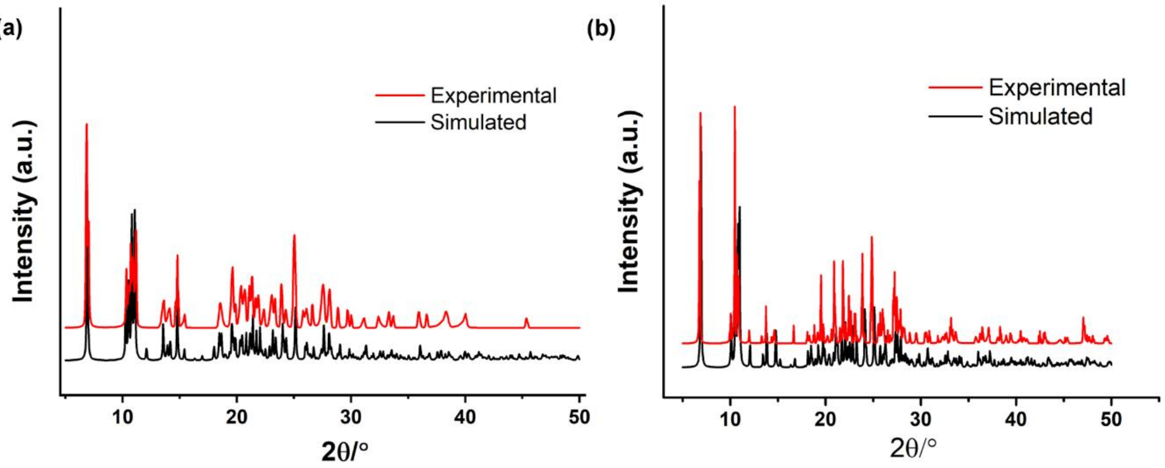

Figure S4: (a,b) PXRD patterns of **1**, **2.**

Table S4: Initial orientations for torque magnetometry of **1** and **2.**

	Orientation	Rotation axis			Magnetic field at θ = 0°		
		a	b′	c*	a	b′	c*
**1**	Rotation 1	0.90693	0.35582	0.21226	0.22066	0.01929	–0.97516
	Rotation 2	–0.35107	0.93124	–0.06102	0.22066	0.01929	–0.97516
	Rotation 3	0.22066	0.01929	–0.97516	–0.90693	–0.35582	–0.21226
**2**	Rotation 1	–0.90103	–0.40183	0.16333	0.26010	–0.19921	0.94481
	Rotation 2	0.34711	–0.89379	–0.28401	–0.90103	–0.40183	0.16333
	Rotation 3	–0.26010	0.19921	–0.94481	–0.90103	–0.40183	0.16333

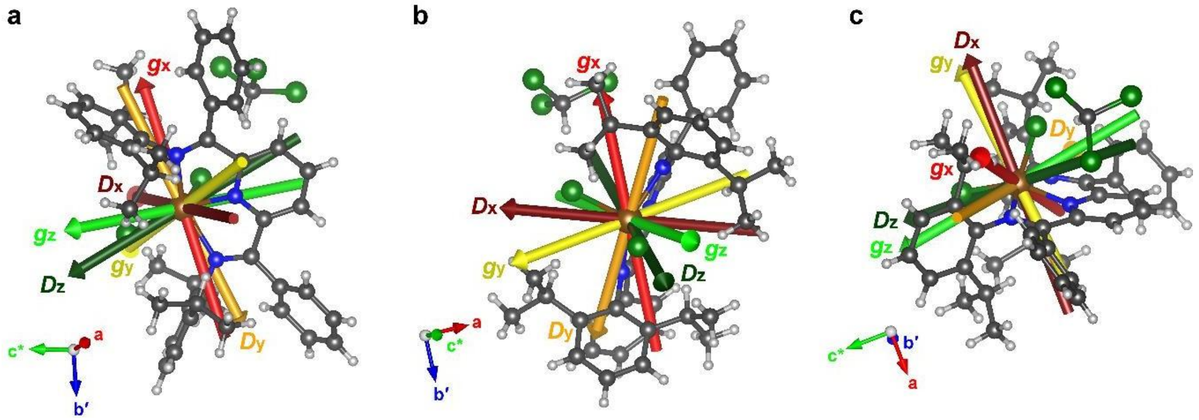

Figure S5: Initial orientations of **1** for (a) rotation 1, (b) rotation 2 and (c) rotation 3, showing experimental principal directions for *g* and *D*. Rotation axis is coming out of the page, initial magnetic field direction is up. Atom colour code: Fe (brown), Cl (green), N (blue), C (dark grey), H (light grey). Vector colour code: *D* easy axis (dark green), *D* intermediate axis (orange), *D* hard axis (maroon), *g* easy axis (light green), *g* intermediate axis (yellow), *g* hard axis (red).

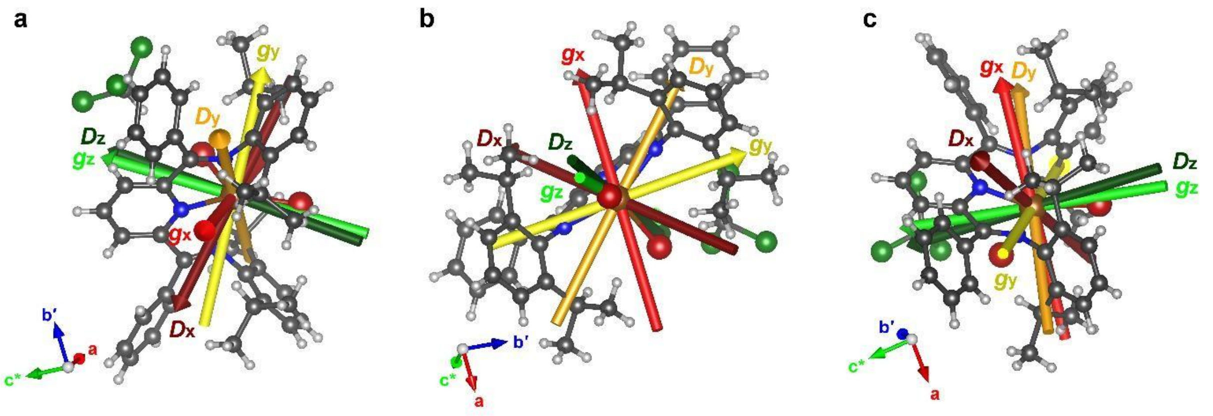

Figure S6: Initial orientations of **2** for (a) rotation 1, (b) rotation 2 and (c) rotation 3, also showing experimental principal directions for *g* and *D*. Rotation axis is coming out of the page, initial magnetic field direction is up. Atom colour code: Fe (brown), Br (maroon), Cl (green), N (blue), C (dark grey), H (light grey). Vector colour code: *D* easy axis (dark green), *D* intermediate axis (orange), *D* hard axis (maroon), *g* easy axis (light green), *g* intermediate axis (yellow), *g* hard axis (red).

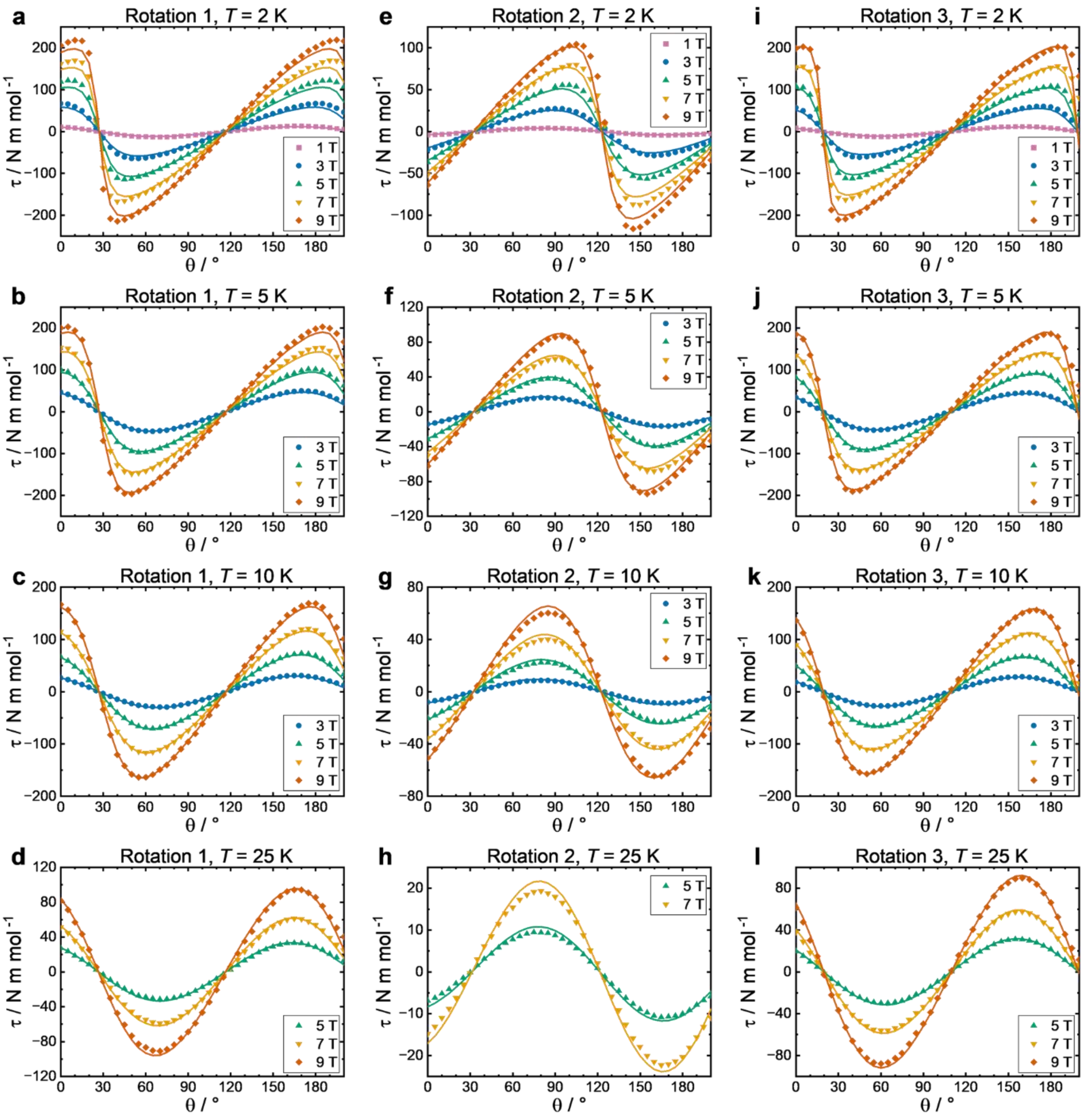

Figure S7: Torque measurements on **1** at 2, 5, 10 and 25 K for rotation 1 (a-d), rotation 2 (e-h) and rotation 3 (i-l), Solid lines indicate simulations.

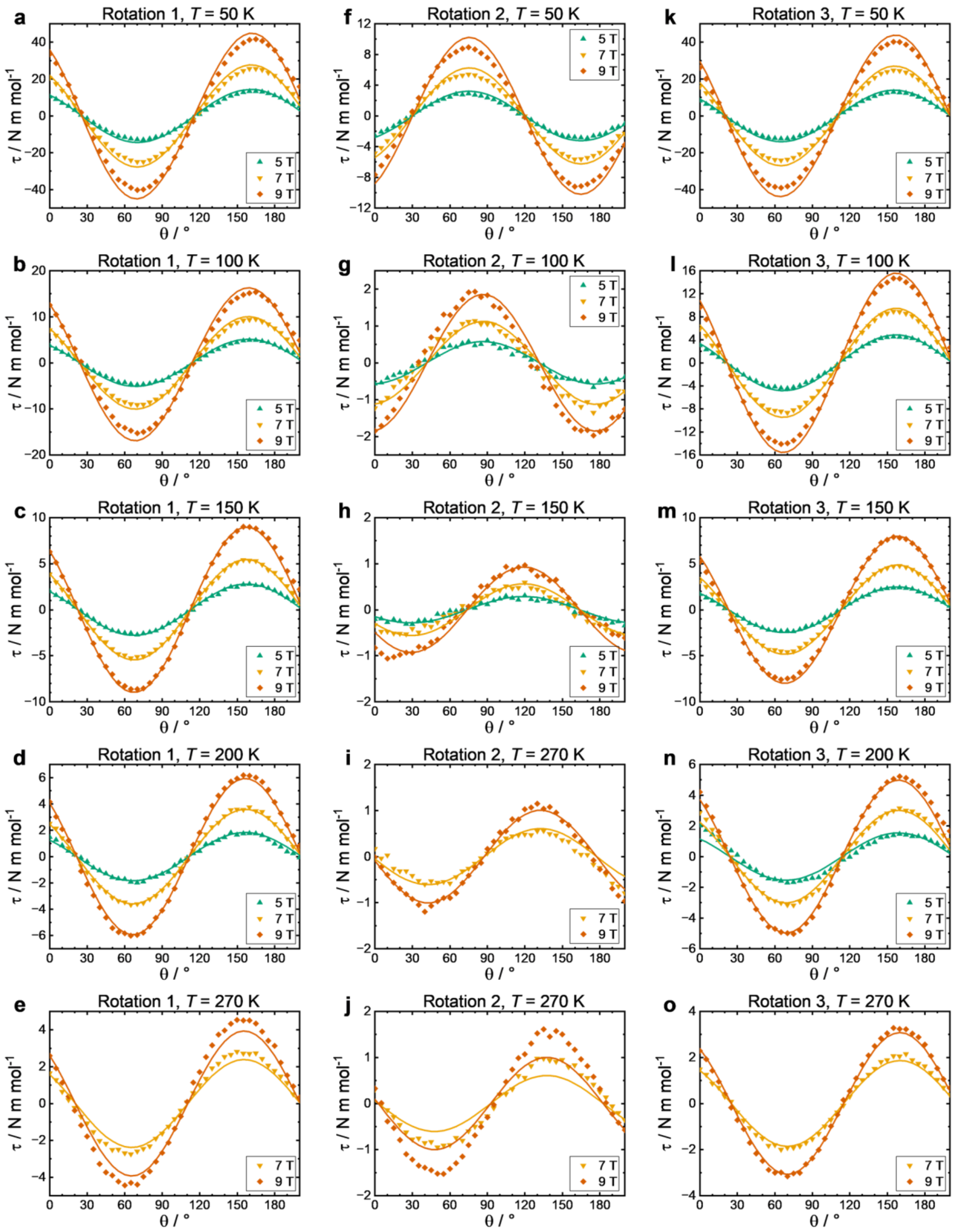

Figure S8: Torque measurements on **1** at 50, 100, 150, 200 and 270 K for rotation 1 (a-e), rotation 2 (f-j) and rotation 3 (k-o). Solid lines indicate simulations.

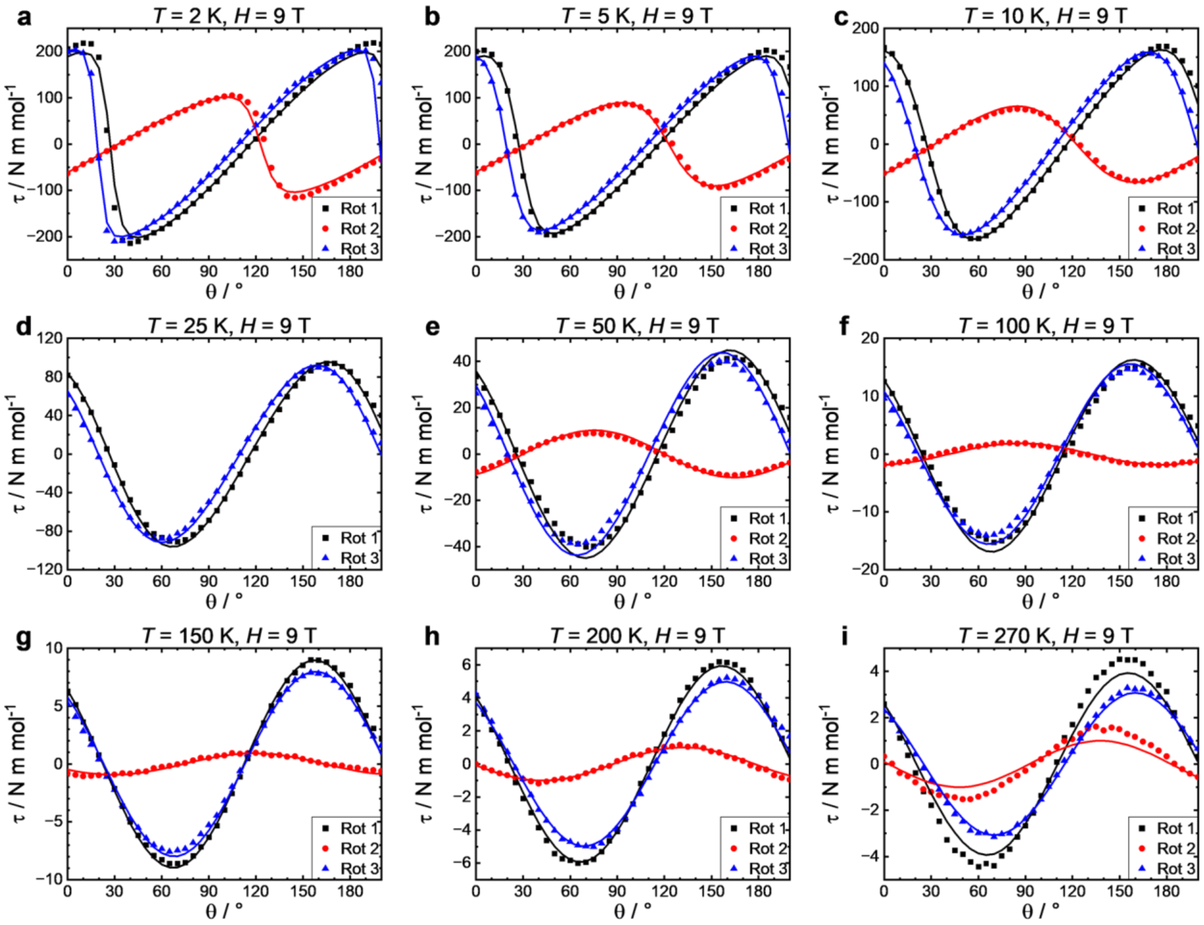

Figure S9: Torque measurements on **1** at 9 T and temperatures of 2–270 K, comparing the three rotations, Solid lines indicate simulations.

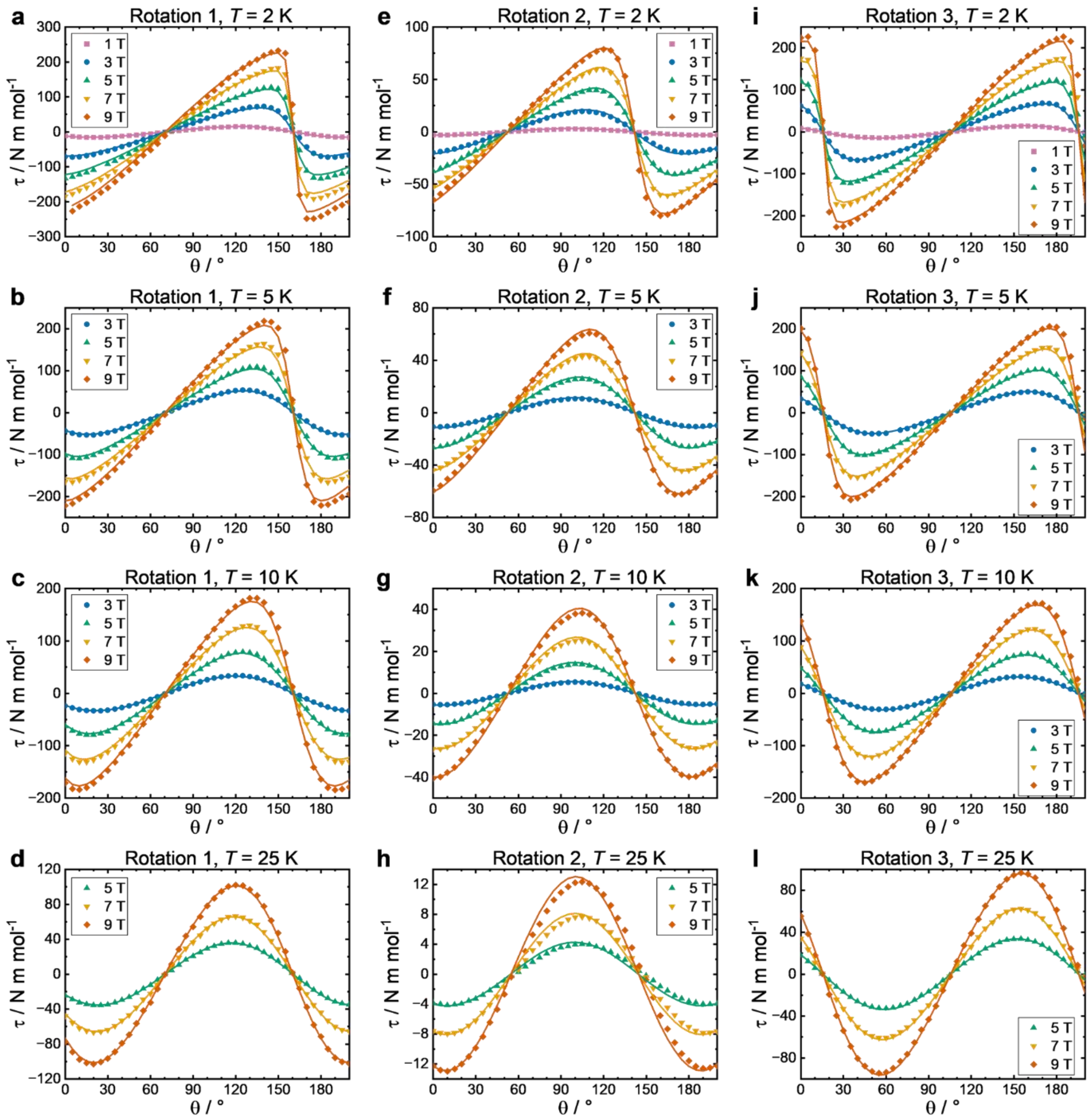

Figure S10: Torque measurements on **2** at 2, 5, 10 and 25 K for rotation 1 (a-d), rotation 2 (e-h) and rotation 3 (i-l). Solid lines indicate simulations.

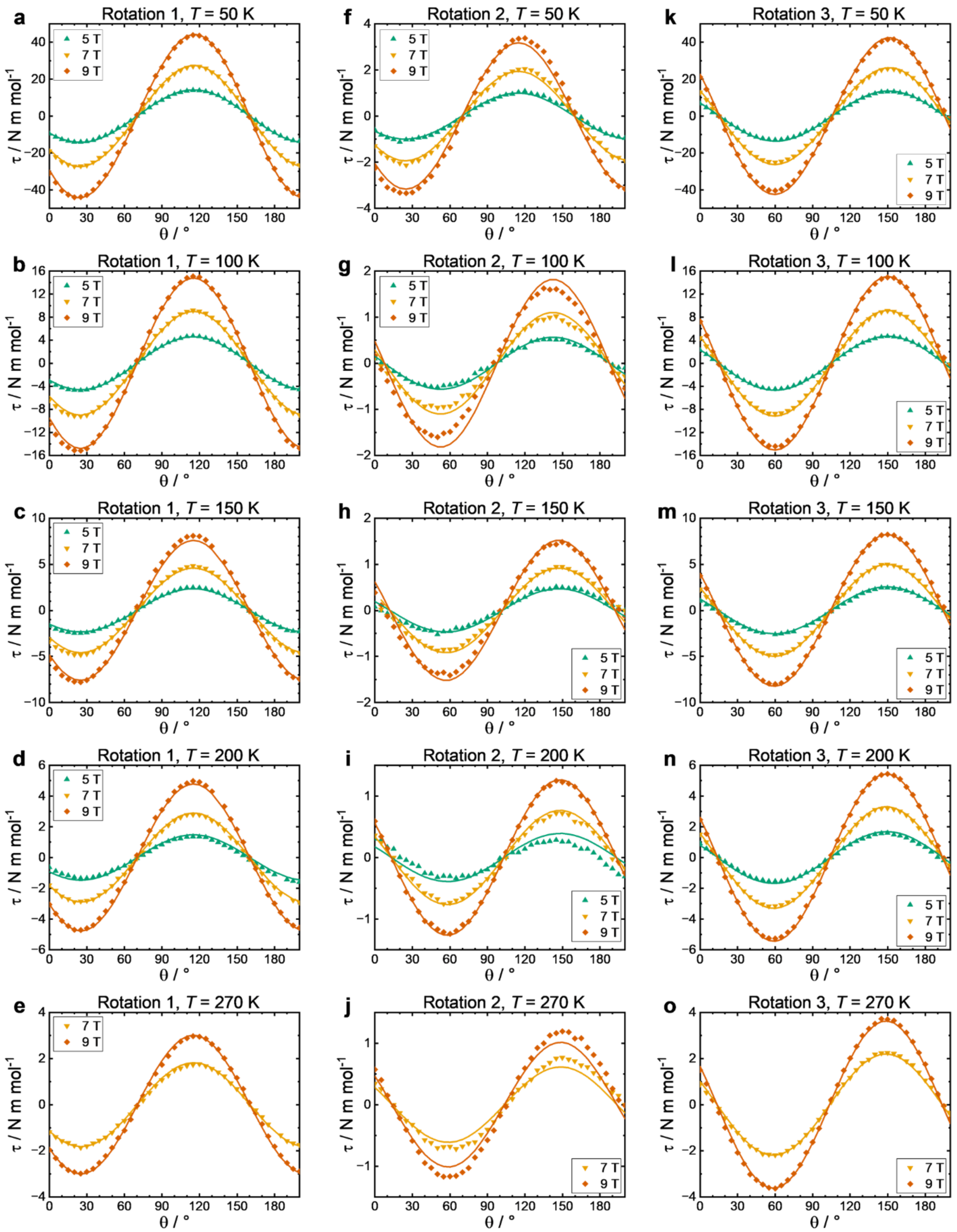

Figure S11: Torque measurements on **2** at 50, 100, 150, 200 and 270 K for rotation 1 (a-e), rotation2 (f-j) and rotation 3 (k-o), Solid lines indicate simulations.

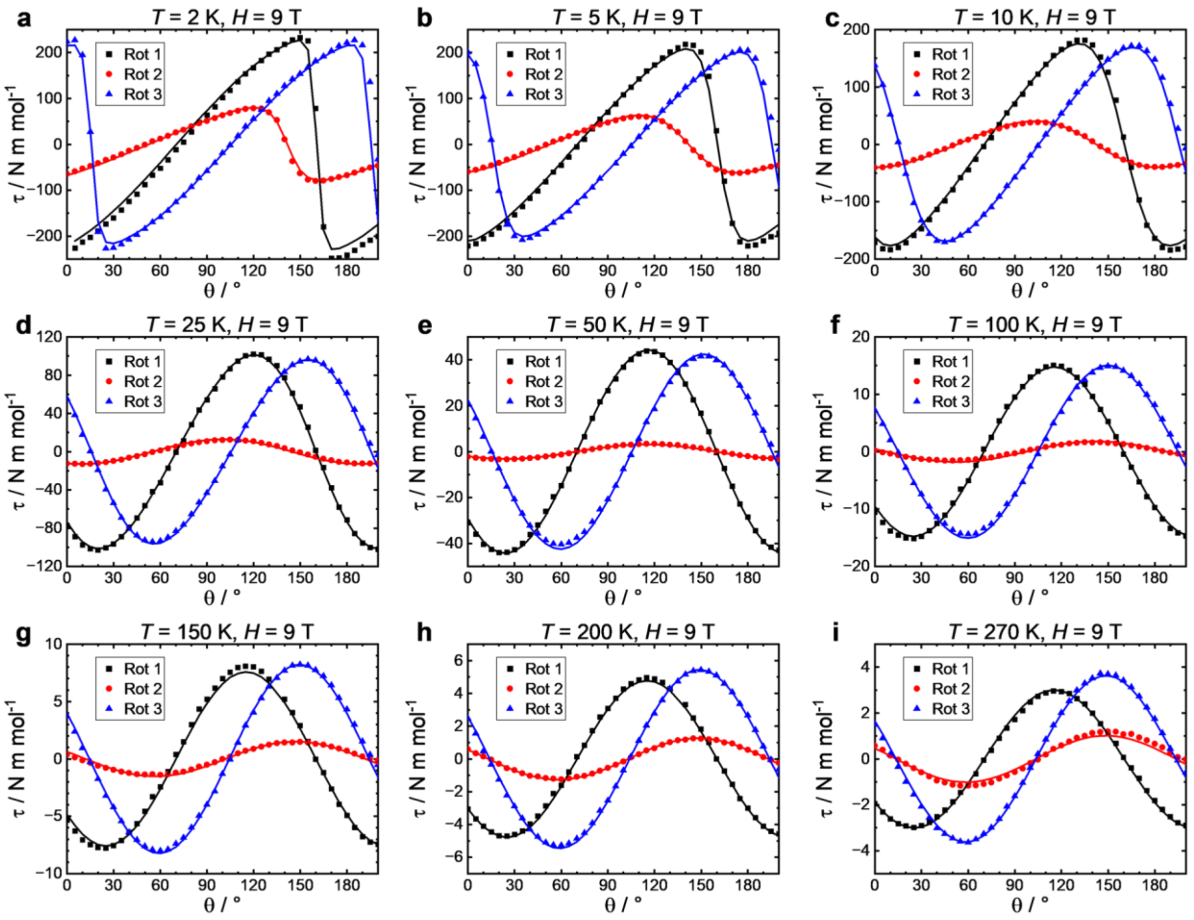

Figure S12: Torque measurements on **2** at 9 T and temperatures of 2–270 K, comparing the three rotations, Solid lines indicate simulations.

Table S5: Orientations of *D* and *g* tensors from torque magnetometry for **1** and **2.**

		D-tensor			*g*-tensor		
		a	b′	c*	a	b′	c*
**1**	X	-0.95373	0.01072	-0.30048	-0.08364	0.22916	-0.96979
	Y	-0.26313	-0.51331	0.81687	-0.99038	0.08850	0.10633
	Z	-0.14549	0.85814	0.49238	0.11019	0.96936	0.21955
**2**	X	-0.69054	0.39181	-0.60798	-0.99259	-0.11161	-0.04806
	Y	-0.72289	-0.34585	0.59818	0.01502	-0.50515	0.86290
	Z	0.02410	0.85257	0.52205	-0.12059	0.85579	0.50308

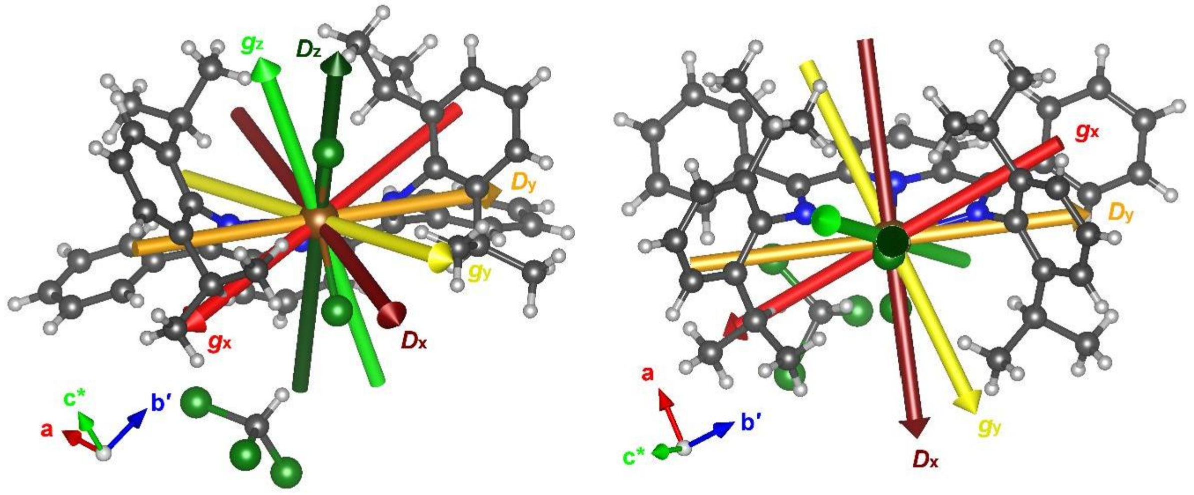

Figure S13: Experimental principal directions for *g* and *D* in **1**. Atom colour code: Fe (brown), Cl (green), N (blue), C (dark grey), H (light grey). Vector colour code: *D* easy axis (dark green),*D* intermediate axis (orange), *D* hard axis (maroon), *g* easy axis (light green), *g* intermediate axis (yellow), *g* hard axis (red).

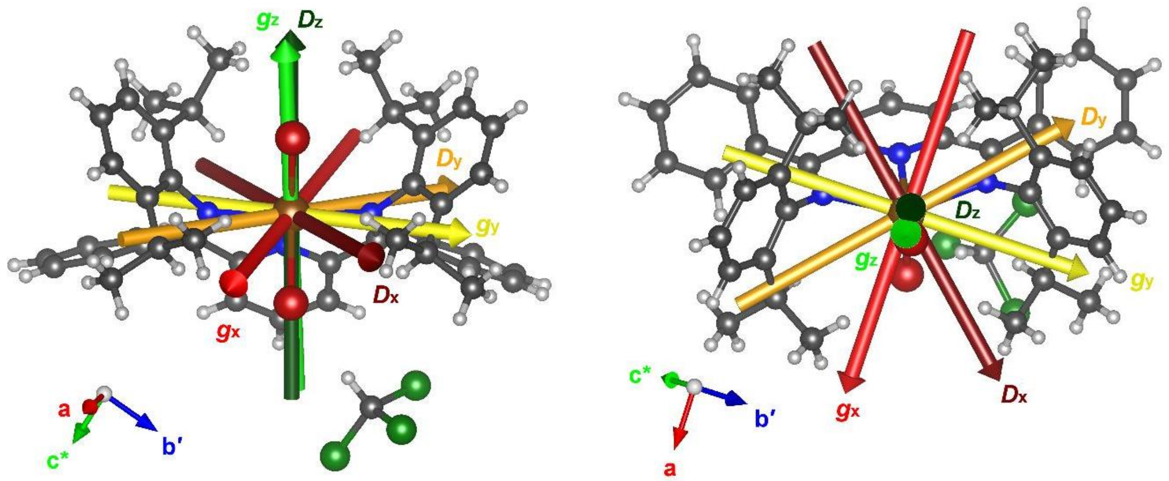

Figure S14: Experimental principal directions for *g* and *D* in **2**. Atom colour code: Fe (brown), Br (maroon), Cl (green), N (blue), C (dark grey), H (light grey). Vector colour code: *D* easy axis (dark green), *D* intermediate axis (orange), *D* hard axis (maroon), *g* easy axis (light green), *g* intermediate axis (yellow), *g* hard axis (red).

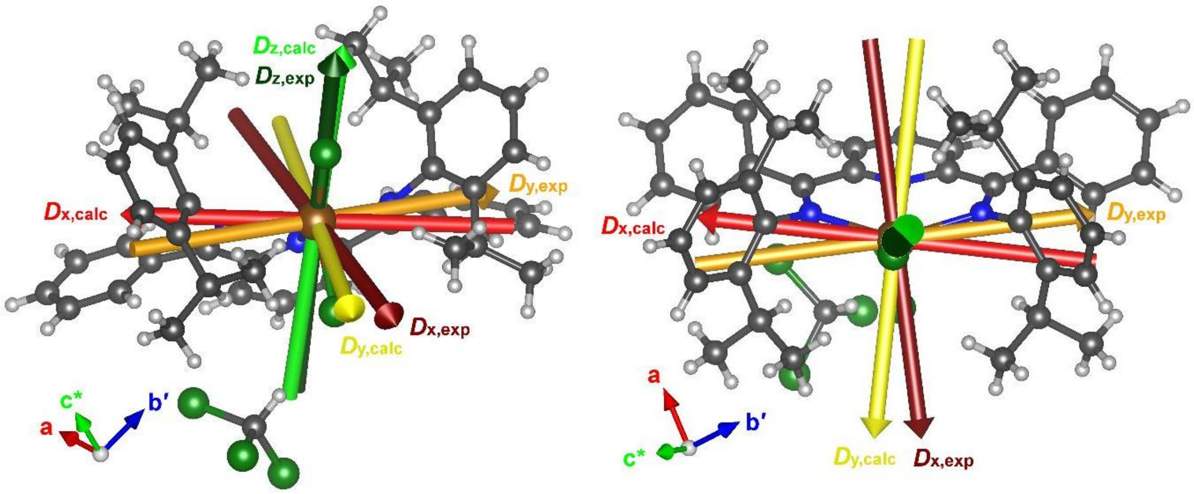

Figure S15: Comparison of experimental and NEVPT2 *D* principal directions for **1**. Atom colour code: Fe (brown), Cl (green), N (blue), C (dark grey), H (light grey). Vector colour code: experimental easy axis (dark green), experimental intermediate axis (orange), experimental hard axis (maroon), NEVPT2 easy axis (light green), NEVPT2 intermediate axis (yellow), NEVPT2 hard axis (red).

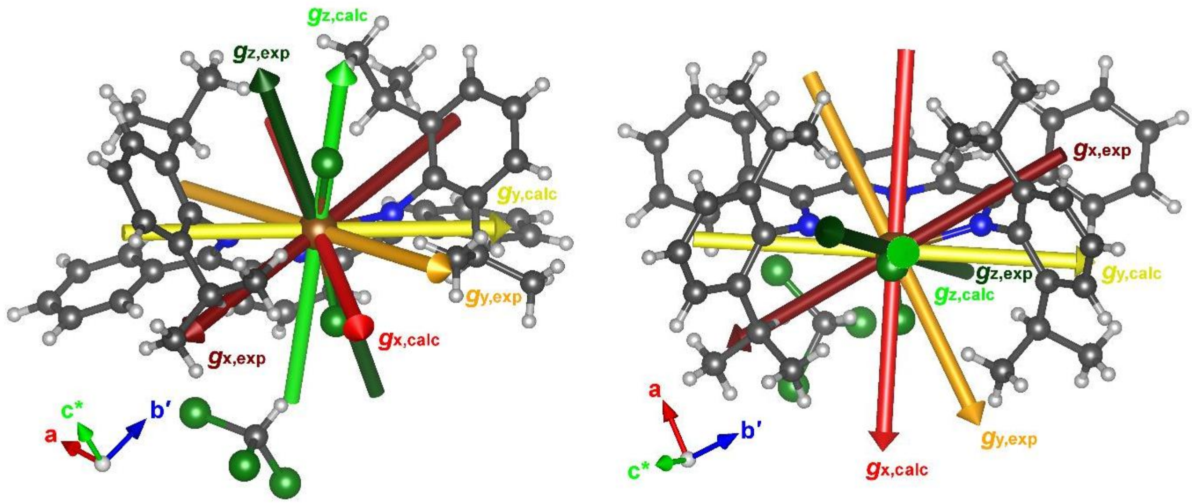

Figure S16: Comparison of experimental and NEVPT2 *g* principal directions for **1**. Atom colour code: Fe (brown), Cl (green), N (blue), C (dark grey), H (light grey). Vector colour code: experimental easy axis (dark green), experimental intermediate axis (orange), experimental hard axis (maroon), NEVPT2 easy axis (light green), NEVPT2 intermediate axis (yellow), NEVPT2 hard axis (red).

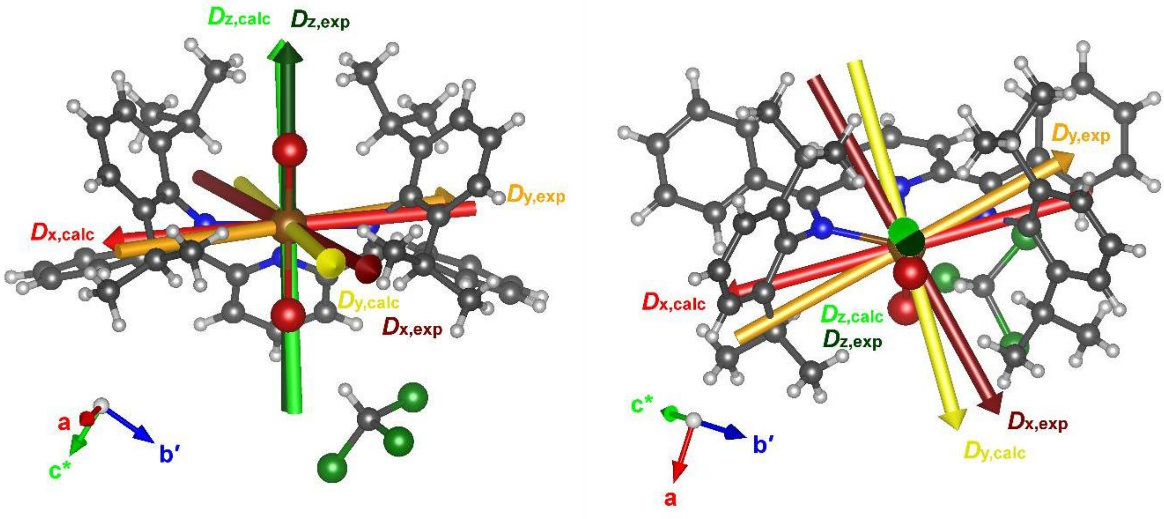

Figure S17: Comparison of experimental and NEVPT2 D principal directions for **2**. Atom colourcode: Fe (brown), Br (maroon), Cl (green), N (blue), C (dark grey), H (light grey). Vector colourcode: experimental easy axis (dark green), experimental intermediate axis (orange), experimental hard axis (maroon), NEVPT2 easy axis (light green), NEVPT2 intermediate axis(yellow), NEVPT2 hard axis (red).

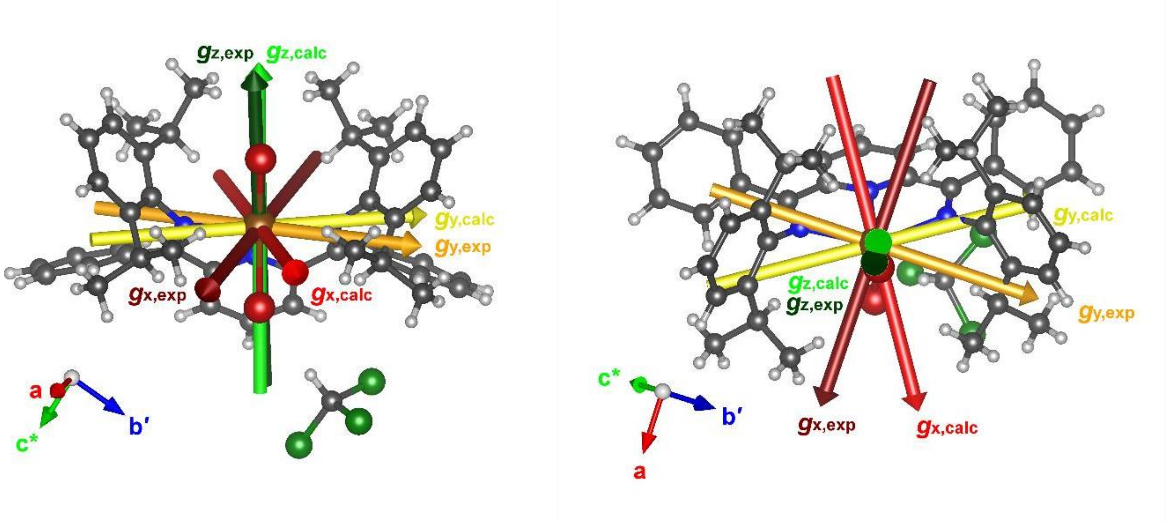

Figure S18: Comparison of experimental and NEVPT2 g principal directions for **2**. Atom colourcode: Fe (brown), Br (maroon), Cl (green), N (blue), C (dark grey), H (light grey). Vector colourcode: experimental easy axis (dark green), experimental intermediate axis (orange), experimental hard axis (maroon), NEVPT2 easy axis (light green), NEVPT2 intermediate axis(yellow), NEVPT2 hard axis (red).

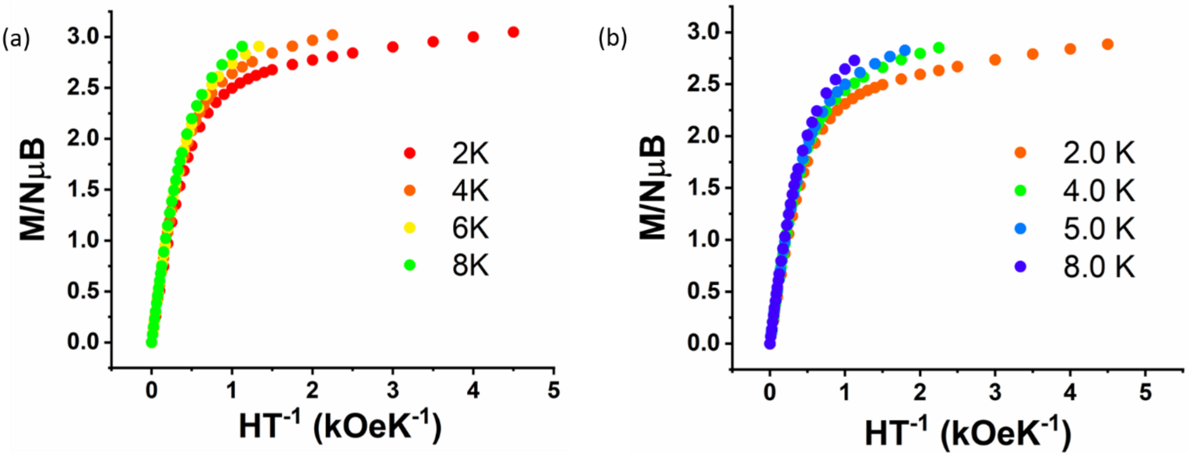

Figure S19: Reduced magnetization data of **1**,**2.**

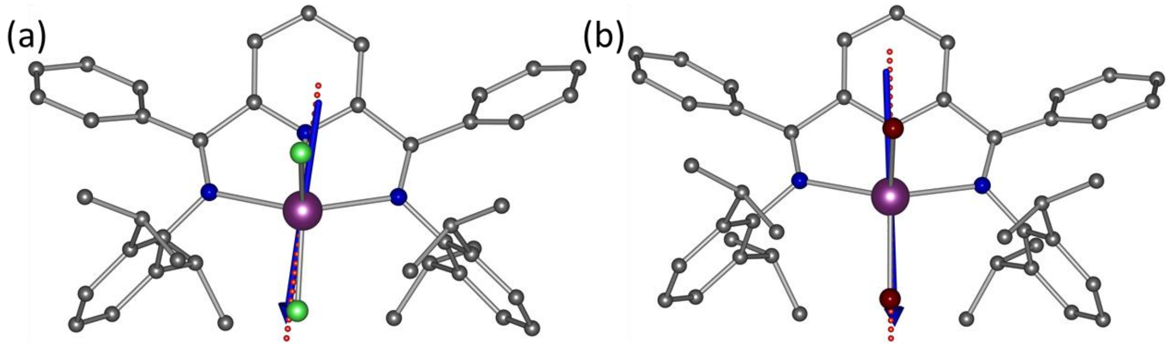

Figure S20: Orientation of calculated NEVPT2 D_z and g_z anisotropic axes for (a) Complex **1**, and (b) Complex **2**. The dark blue arrows represent the D_z directions, while the red dotted lines indicate the g_z directions. The angles between the D_z and g_z directions from the Fe atoms are 3.626°, and 4.485° for Complexes **1**-**2**, respectively

Table S6: Spin Hamiltonian parameters derived from CASSCF/NEVPT2 level of theory for complexes **1** and **2**.

**Parameters**	**1**		**2**	
	**CASSCF**	**NEVPT2**	**CASSCF**	**NEVPT2**
**D**	-27.81	-29.87	-31.03	-29.78
**|E/D|**	0.317	0.316	0.230	0.232
g_x	1.932	1.902	1.917	1.925
g_y	2.250	2.247	2.114	2.120
g_z	2.641	2.652	2.661	2.628

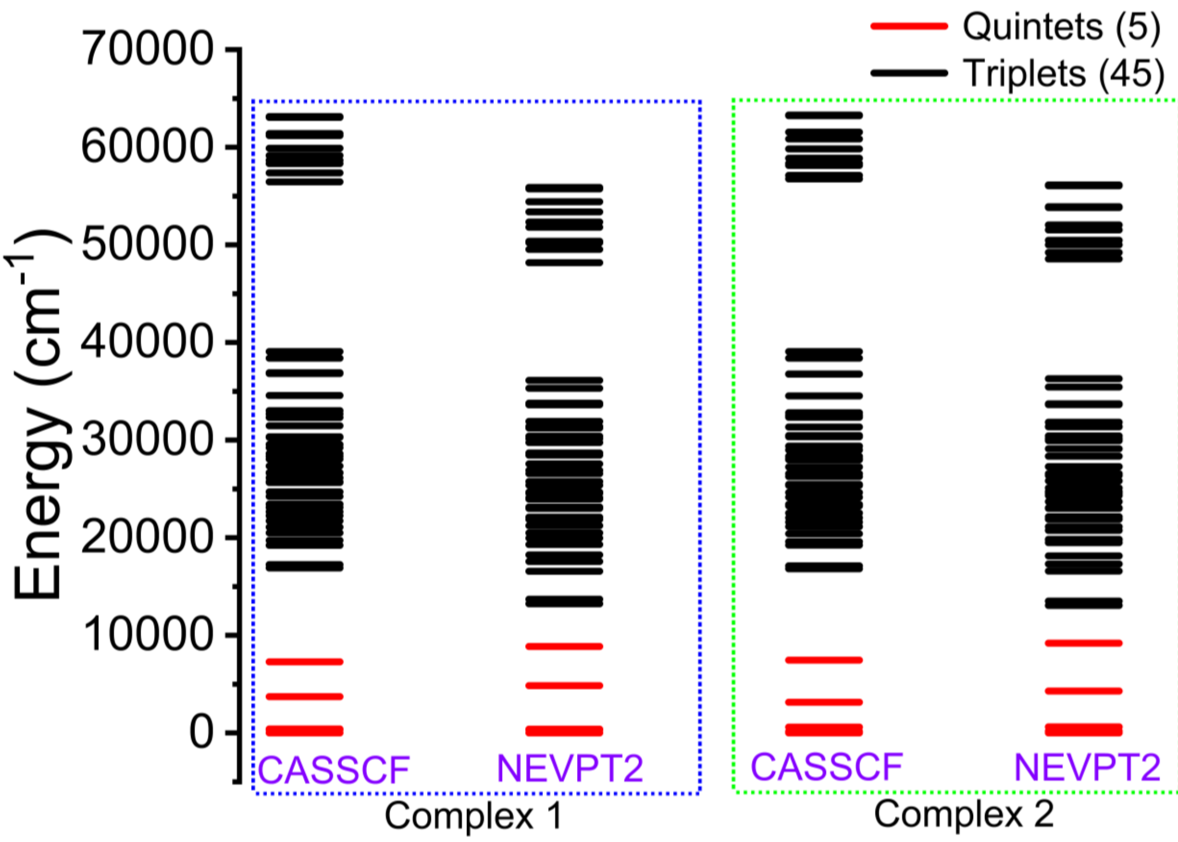

Figure 21: CASSCF/NEVPT2 multiplet energies for complexes **1** and **2**.

Table S7: Norms of the projected states together with the CASSCF-derived multiplet energies obtained from the effective Hamiltonian ZFS analysis.

Complex **1**				Complex **2**			
Multiplicity	Root	E (cm^{-1})	S (norm)	Mul	Root	E (cm^{-1})	S (norm)
5	1	0.0	0.999	5	1	0.0	0.994

5	2	329.0	0.883	5	2	164.4	0.993
5	3	411.8	0.882	5	3	328.8	0.999
5	4	3734.7	0.999	5	4	2847.0	0.999
5	5	7285.5	0.999	5	5	6157.2	1.000
3	1	16915.7	1.000	3	1	15547.7	1.000
3	2	17246.6	0.999	3	2	15714.5	0.999
3	3	19215.2	0.999	3	3	18086.6	0.998
:	:	:	:	:	:	:	:
:	:	:	:	:	:	:	:

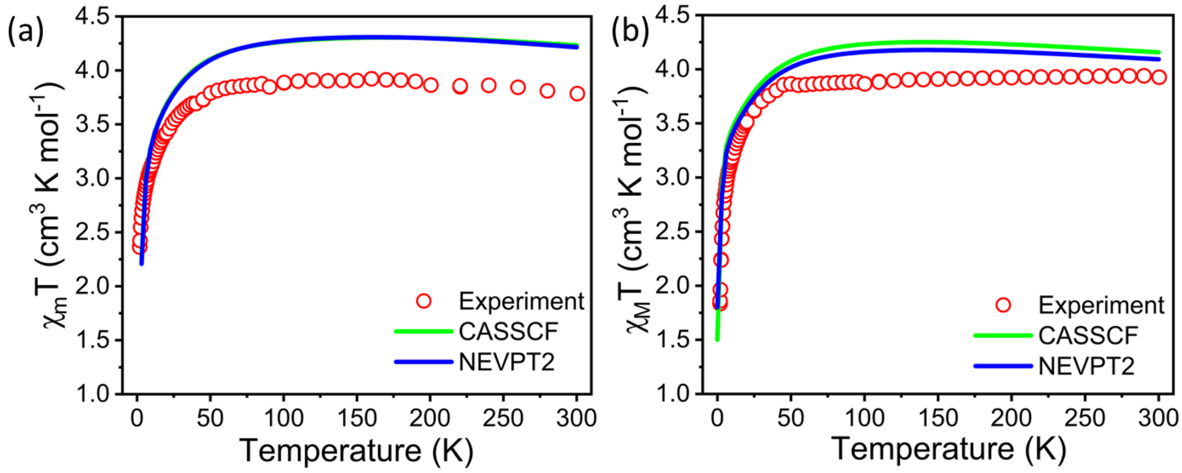

Figure S22: (a, b) Comparison of observed magnetic susceptibility with calculated values obtained from CASSCF and NEVPT2 calculations for **1** and **2** respectively. Red symbols represent the experimental magnetic susceptibility, while the blue line represents NEVPT2 calculations, and the light green line represents CASSCF magnetic susceptibility.

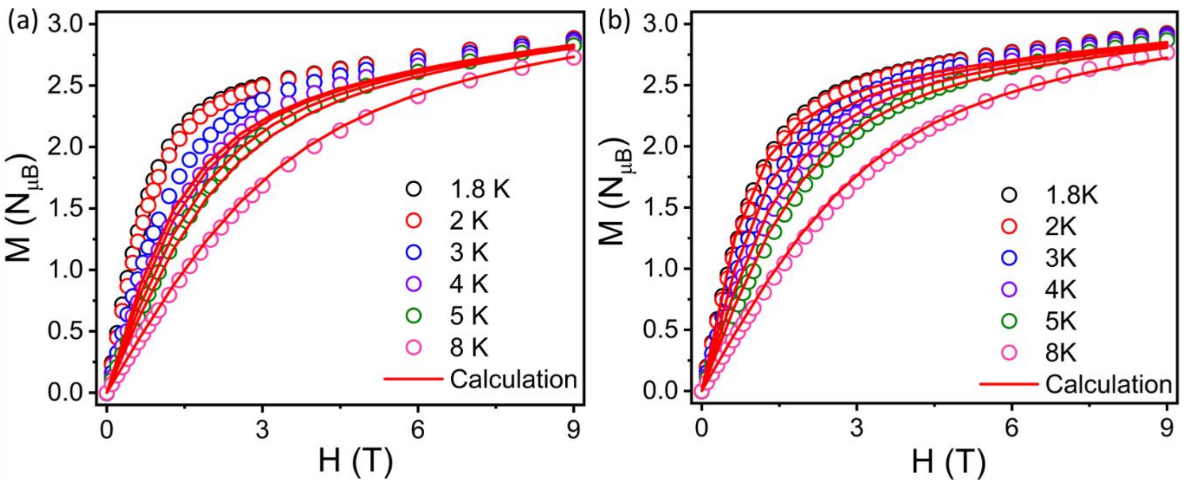

Figure S23: Comparison of observed isothermal field-dependent magnetisation measurements with NEVPT2 calculations for (a) **1** and (b) **2**. Experimental magnetisation at 1.8 K (black), 2 K (red), 3 K (blue), 4 K (violet), 5 K (green), and 8 K (pink) is represented by symbols, while the red solid line at each temperature shows the calculated magnetisation from NEVPT2.

Table S8: NEVPT2 transition energies, their corresponding wavefunctions, and their individual contributions towards D and E values for the ground state and the first five excited states of Complex **1**.

**Spin free energy state of complex 1**	**Major CASSCF electronic configuration**	**NEVPT2 transition energy (cm^{-1})**	**Contribution to D (cm^{-1})**	**Contribution to E (cm^{-1})**
**GS**	$(d_{xz})^2(d_{yz})^1(d_{xy})^1(d_{z^2})^1(d_{x^2-y^2})^1$ (57%)<br>$(d_{xz})^1(d_{yz})^1(d_{xy})^1(d_{z^2})^1(d_{x^2-y^2})^2$ (21%)<br>$(d_{xz})^1(d_{yz})^2(d_{xy})^1(d_{z^2})^1(d_{x^2-y^2})^1$ (21%)	0.0	0.0	0.0
**1st ES**	$(d_{xz})^1(d_{yz})^2(d_{xy})^1(d_{z^2})^1(d_{x^2-y^2})^1$ (90%)<br>$(d_{xz})^1(d_{yz})^1(d_{xy})^1(d_{z^2})^2(d_{x^2-y^2})^1$ (6%)	329.0	-34.799	-1.041
**2nd ES**	$(d_{xz})^1(d_{yz})^1(d_{xy})^2(d_{z^2})^1(d_{x^2-y^2})^1$ (51%)<br>$(d_{xz})^1(d_{yz})^1(d_{xy})^1(d_{z^2})^2(d_{x^2-y^2})^1$ (31%)	411.8	3.693	-8.892
**3rd ES**	$(d_{xz})^1(d_{yz})^1(d_{xy})^1(d_{z^2})^2(d_{x^2-y^2})^1$ (39%)<br>$(d_{xz})^1(d_{yz})^1(d_{xy})^2(d_{z^2})^1(d_{x^2-y^2})^1$ (37%)<br>$(d_{xz})^1(d_{yz})^1(d_{xy})^1(d_{z^2})^1(d_{x^2-y^2})^2$ (16%)	3734.7	0.665	1.374
**4th ES**	$(d_{xz})^1(d_{yz})^1(d_{xy})^1(d_{z^2})^1(d_{x^2-y^2})^2$ (37%)<br>$(d_{xz})^1(d_{yz})^2(d_{xy})^1(d_{z^2})^1(d_{x^2-y^2})^1$ (29%)	7285.5	-0.681	0.173

Table S9: NEVPT2 transition energies, their corresponding wavefunctions, and their individual contributions towards D and E values for the ground state and the first five excited states of Complex **2**.

**Spin free energy state of complex 2**	**Major CASSCF electronic configuration**	**NEVPT2 transition energy (cm^{-1})**	**Contribution to D (cm^{-1})**	**Contribution to E (cm^{-1})**
**GS**	$(d_{xz})^2(d_{yz})^1(d_{xy})^1(d_{z^2})^1(d_{x^2-y^2})^1$ (57%)<br>$(d_{xz})^1(d_{yz})^1(d_{xy})^2(d_{z^2})^1(d_{x^2-y^2})^1$ (20%)<br>$(d_{xz})^1(d_{yz})^2(d_{xy})^1(d_{z^2})^1(d_{x^2-y^2})^1$ (15%)	0.0	0.0	0.0
**1st ES**	$(d_{xz})^1(d_{yz})^2(d_{xy})^1(d_{z^2})^1(d_{x^2-y^2})^1$ (59%)<br>$(d_{xz})^1(d_{yz})^1(d_{xy})^1(d_{z^2})^2(d_{x^2-y^2})^1$ (22%)	164.4	-36.008	-0.055
**2nd ES**	$(d_{xz})^1(d_{yz})^1(d_{xy})^2(d_{z^2})^1(d_{x^2-y^2})^1$ (37%)<br>$(d_{xz})^1(d_{yz})^1(d_{xy})^1(d_{z^2})^2(d_{x^2-y^2})^1$ (31%)	600.6	6.142	-6.650
**3rd ES**	$(d_{xz})^1(d_{yz})^1(d_{xy})^1(d_{z^2})^2(d_{x^2-y^2})^1$ (43%)<br>$(d_{xz})^1(d_{yz})^1(d_{xy})^1(d_{z^2})^1(d_{x^2-y^2})^2$ (34%)<br>$(d_{xz})^1(d_{yz})^1(d_{xy})^2(d_{z^2})^1(d_{x^2-y^2})^1$ (18%)	3164.7	1.221	1.217
**4th ES**	$(d_{xz})^1(d_{yz})^1(d_{xy})^2(d_{z^2})^1(d_{x^2-y^2})^1$ (34%)<br>$(d_{xz})^1(d_{yz})^1(d_{xy})^1(d_{z^2})^2(d_{x^2-y^2})^1$ (30%)	7465.3	0.319	0.579

Table S10: Ligand field parameters derived from AILFT calculations conducted at the NEVPT2 level of theory for compounds **1**, and **2**. The values of the B, C, and ξ parameters are given in units of cm^{-1}.

**Parameter**	**Free Fe (II)**	**1**	**2**	**% reduction**	
				**1**	**2**
ζ	415.7	398.1	389.6	4.23	6.27
B	1016.1	920.3	921.3	9.45	9.32
C	3958.4	3637.8	3630.3	8.09	8.28
C/B	3.90	3.95	3.94	1.28	1.02

Table S11: All the optimized model complex structures, along with their distances from the basal plane (in Å) and their SH parameters.

Model	Optimized Structure	Distance from the basal plan (Å)	D (cm^{-1})	E/D	g_x, g_y, g_z
a	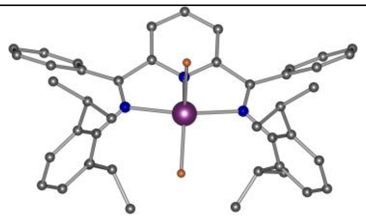 **1-F**	0.789	-10.13	0.046	2.027, 2.037, 2.184
b	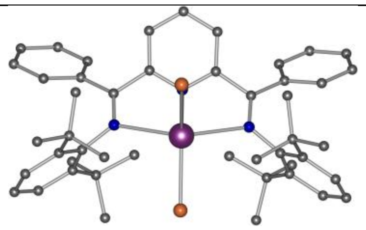 **1-F (^{t}Bu)**	0.802	-11.38	0.033	2.026, 2.035, 2.209
c	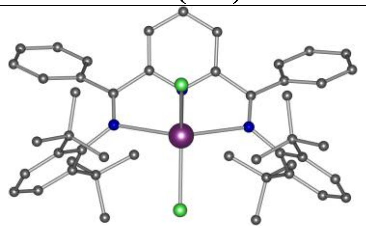 **1-Cl (^{t}Bu)**	0.748	-11.00	0.207	2.039, 2.134, 2.278
d	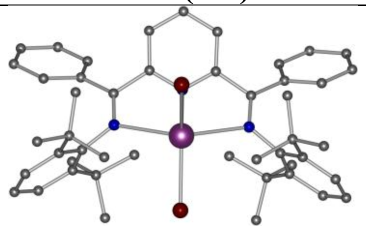 **1-Br (^{t}Bu)**	0.722	15.23	0.088	2.002, 2.294, 2.344
e	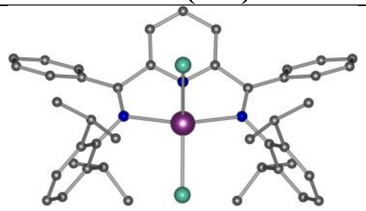 **1-I**	0.636	-42.65	0.077	1.199, 1.358, 2.695

f	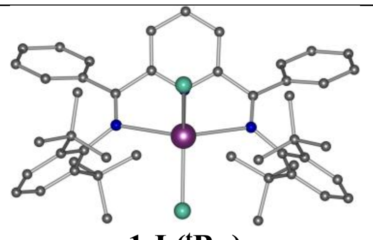 **1-I (^{t}Bu)**	0.714	21.45	0.218	1.905, 2.334, 2.468
g	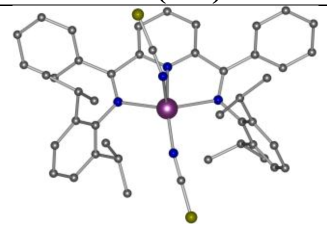 **1-NCS**	0.602	-25.23	0.042	2.010, 2.031, 2.501
h	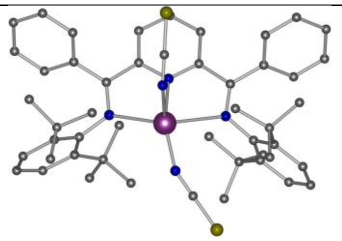 **1-NCS (^{t}Bu)**	0.682	-36.42	0.023	1.976, 2.002, 2.671
i	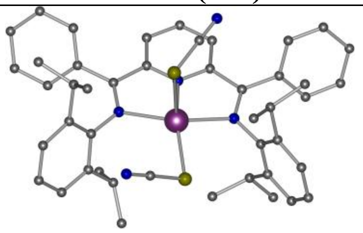 **1-SCN**	0.515	-14.43	0.130	2.014, 2.100, 2.309
j	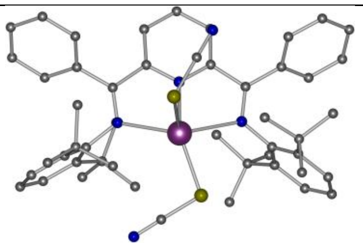 **1-SCN (^{t}Bu)**	0.623	-16.66	0.125	1.999, 2.059, 2.330

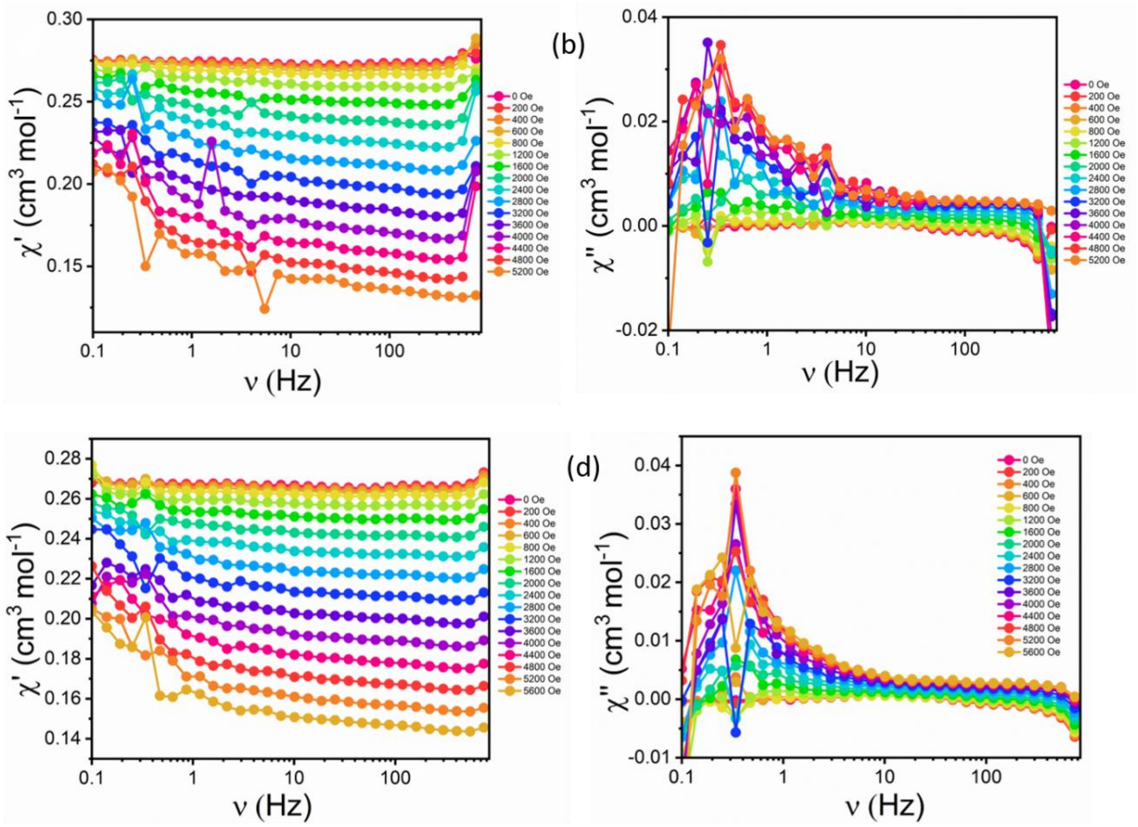

Figure S24: Field-dependent in-phase and out-of-phase susceptibility signals of **1** (a,b), **2** **(**c,d) at 2 K.

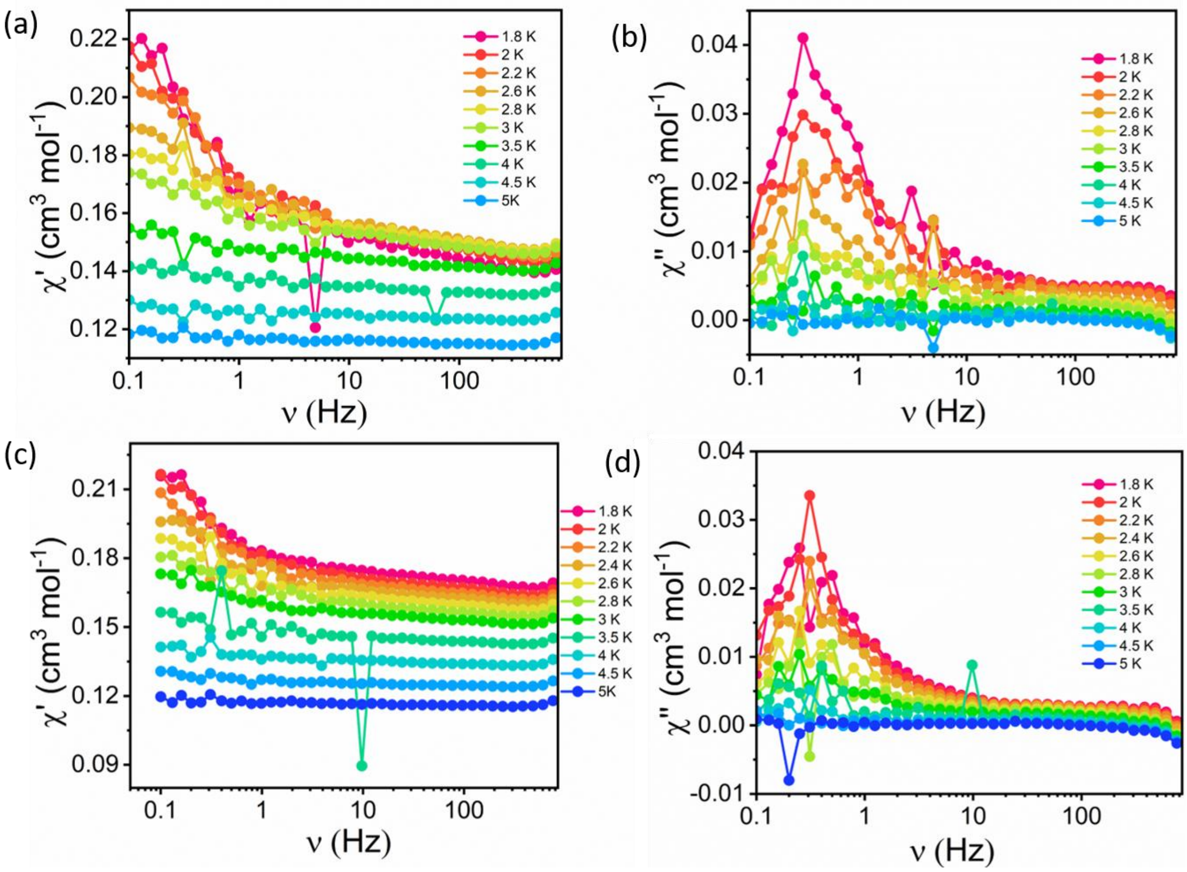

Figure S25: Frequency-dependent in-phase and out-of-phase susceptibility signals of **1** (a,b) and **2** (c,d) at 4.8 kOe DC field.

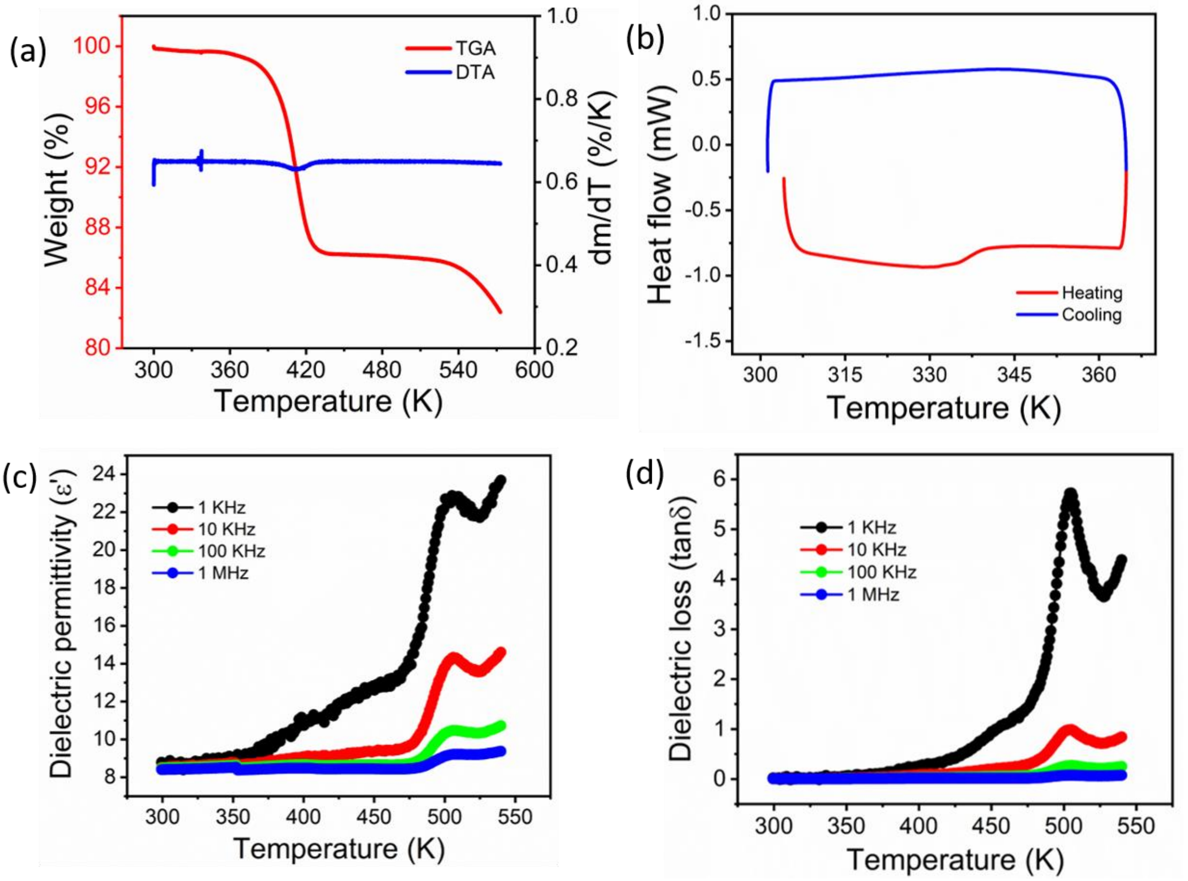

Figure S26. (a) TGA and (b) DSC curves for heating and cooling runs (c,d) temperature dependent dielectric constant (ε') and dielectric loss (tan δ) of **1.**

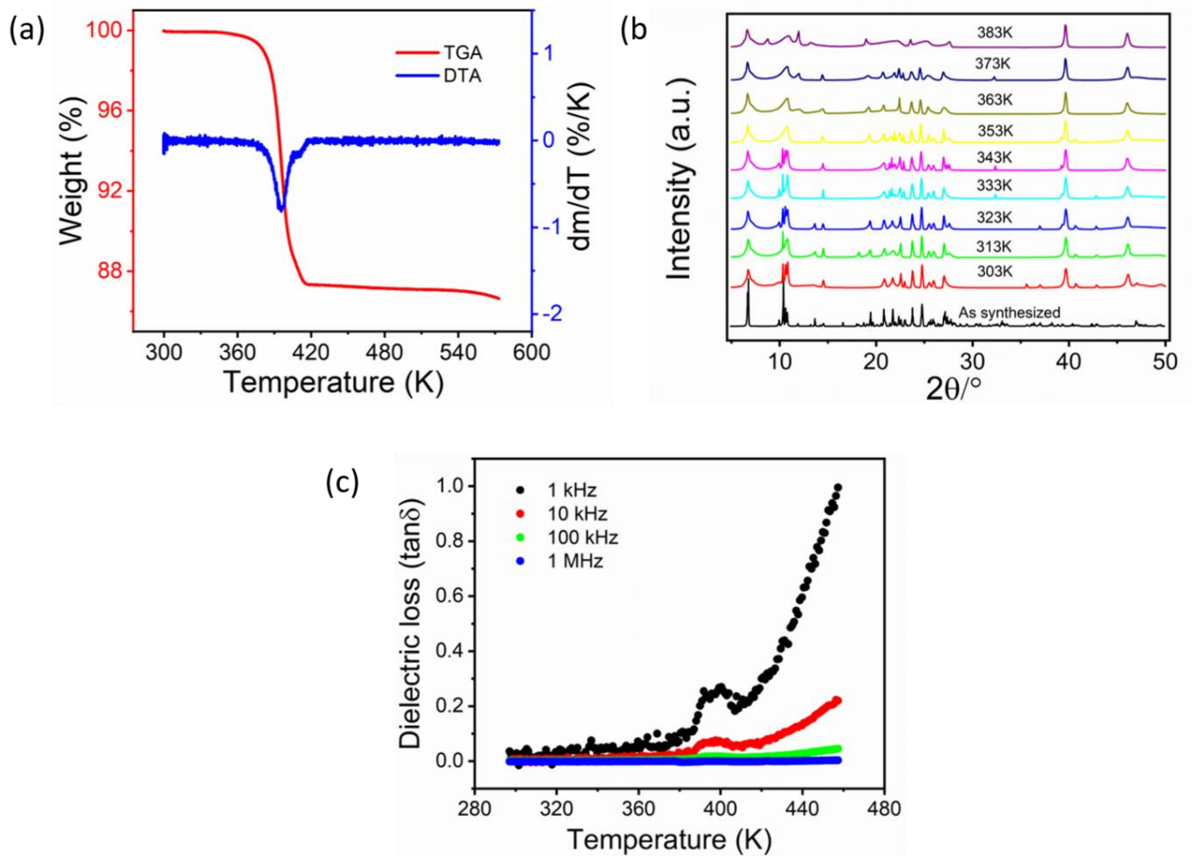

Figure S27: (a) TGA and (b) Dielectric loss (tan δ) of **2.**

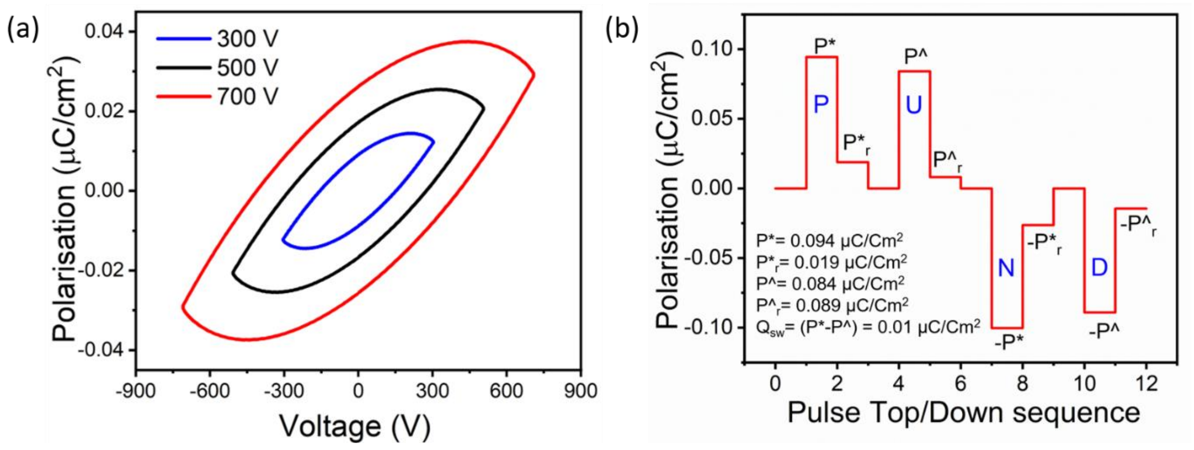

Figure S28: (a) P-E hysteresis loop of **1** (b) PUND (Positive-Up Negative-Down) polarisation measurement of **2** with a pulse width of 50ms and delay time of 200ms.

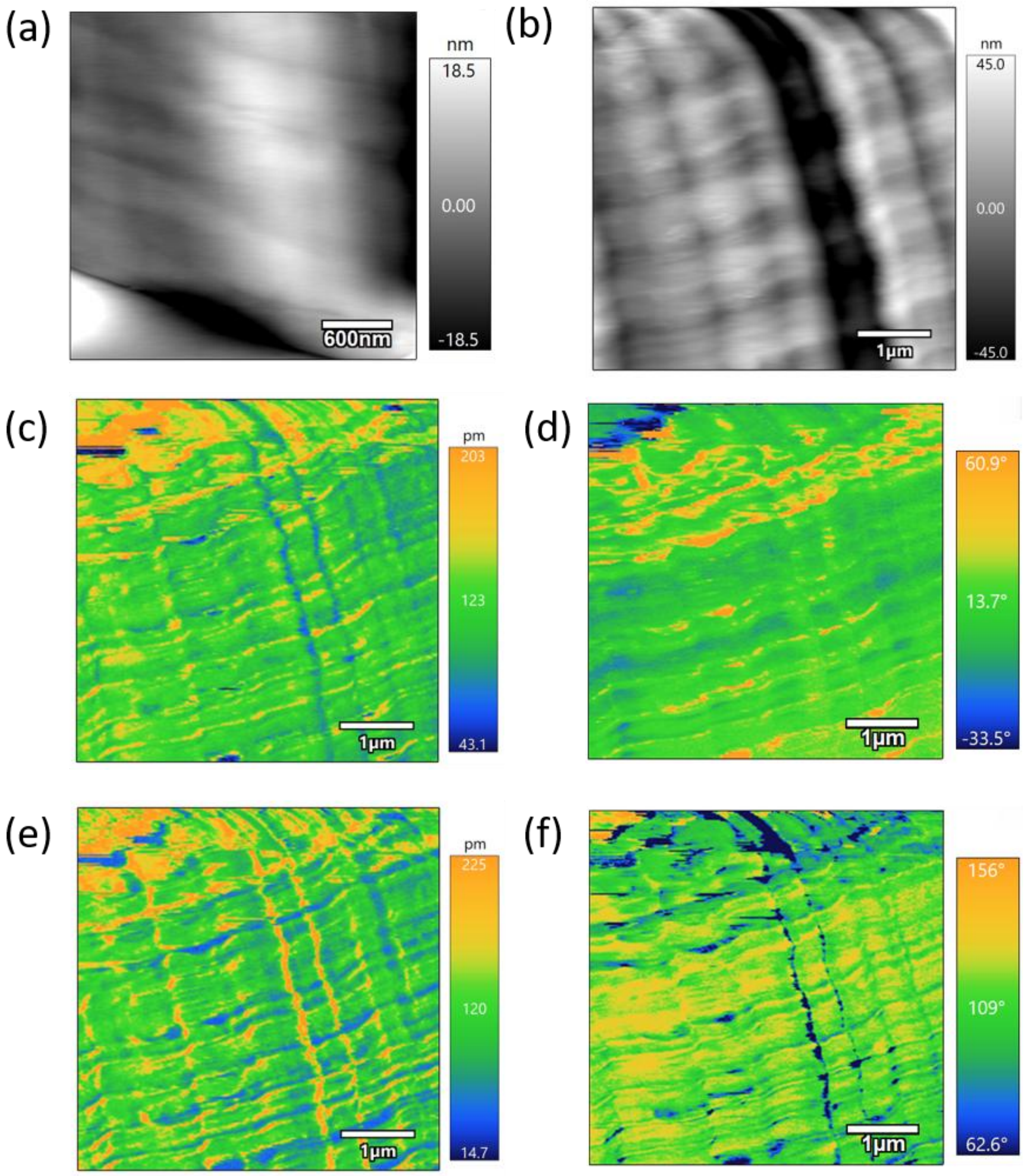

Figure S29. (a,b) Surface topography of **2** and **1** respectively on a single crystal (b) OFF-state amplitude-Voltage butterfly loop and Phase Shift-Voltage hysteresis loop (c, d) Vertical PFM amplitude and phase (e, f) Lateral PFM amplitude and phase showing the different domains.

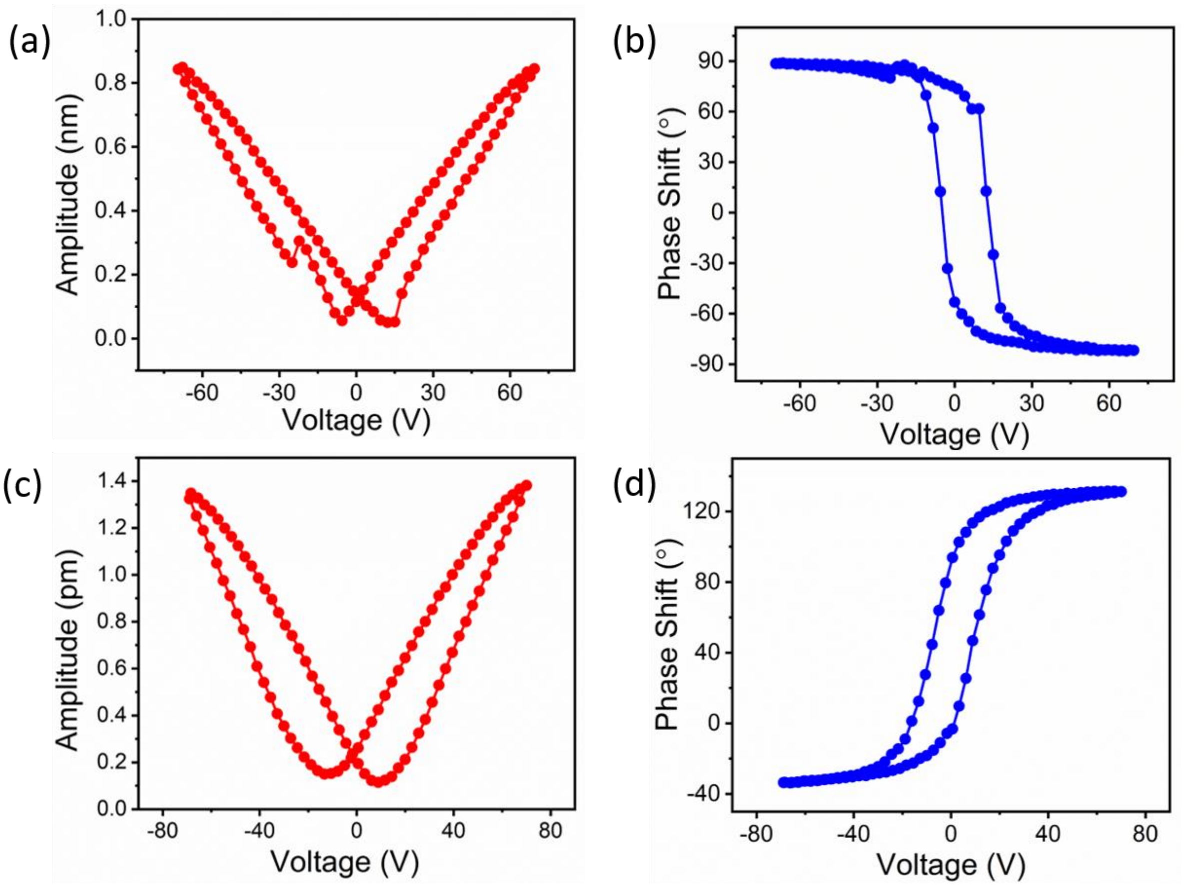

Figure S30. ON-state graph of Amplitude-Voltage butterfly loop & Phase Shift-Voltage hysteresis loop of **1** (a, b) and **2** (c, d) measured on a single crystal.

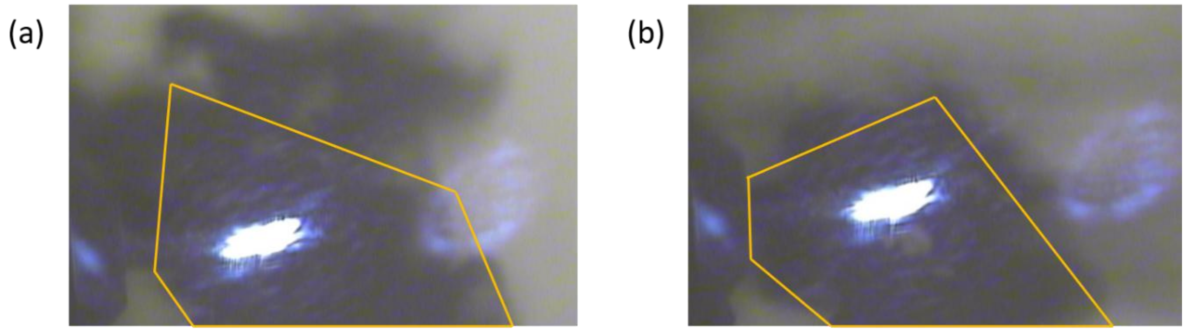

Figure S31. Optical micrograph of **1** and **2** obtained through PFM microscope camera highlighting the entire single-crystal region (outlined) used for PFM measurements respectively.

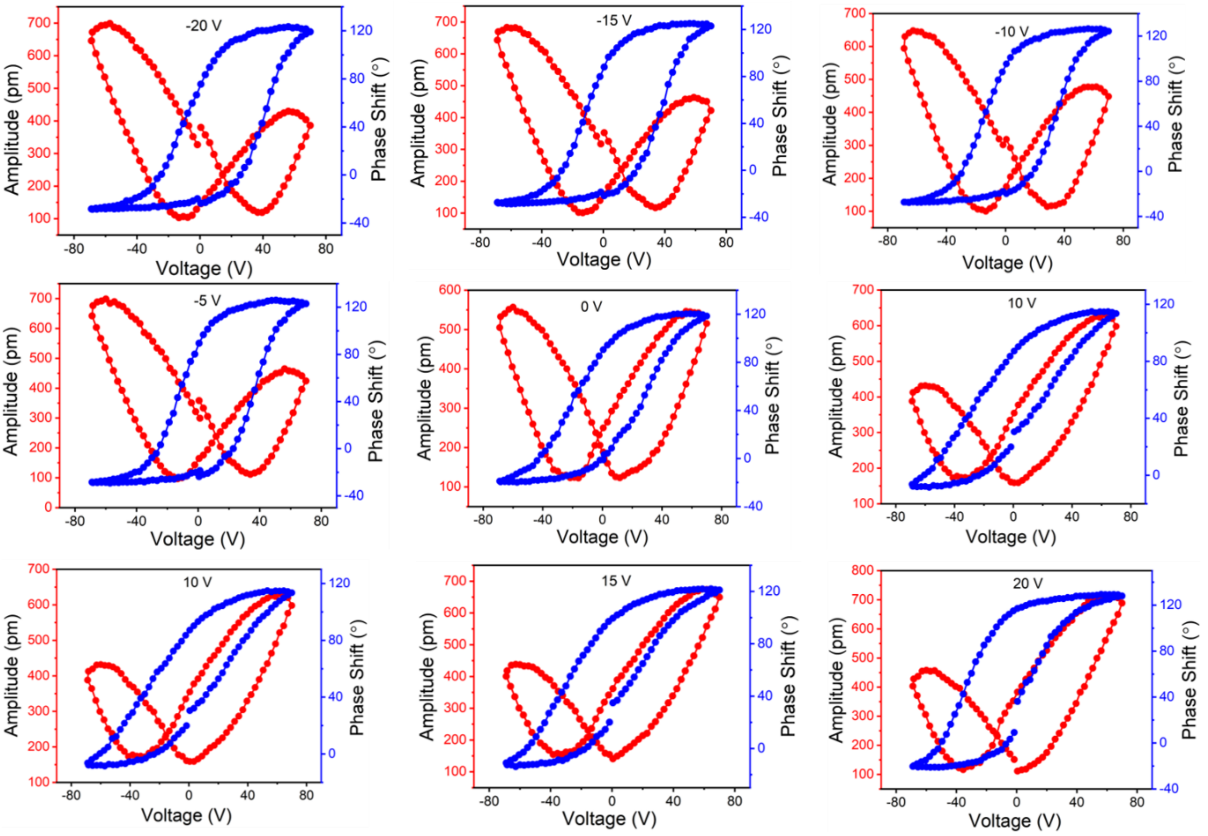

Figure S32. OFF-state PFM amplitude and phase loop of **2** measured at -20 V, -15 V, -10 V, -5 V, 0 V, +5 V, +10 V, +15 V, +20 V of tip voltages.

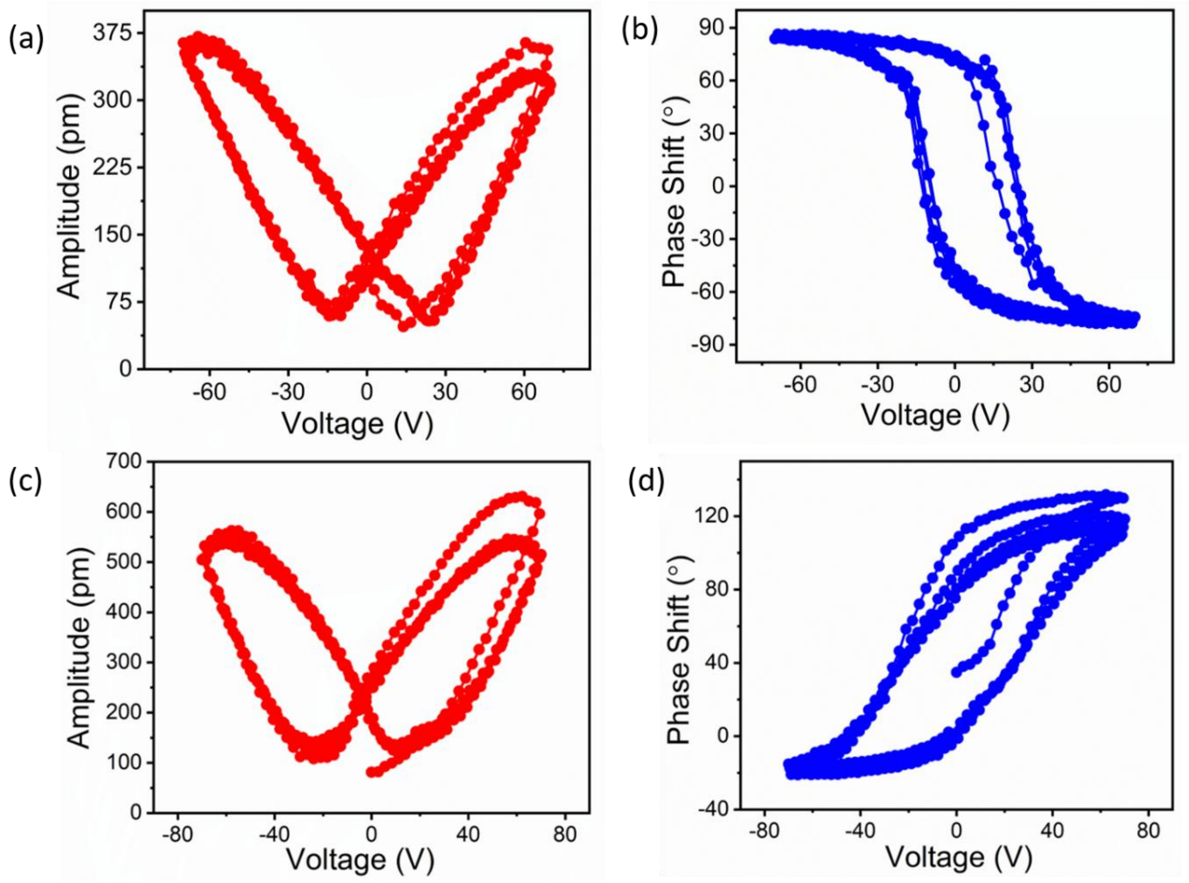

Figure S33. OFF-state PFM amplitude and phase hysteresis loop of **1**(a, b) and **2**(a, b) measured for multiple cycles.

References

1. Jurca, T.; Dawson, K.; Mallov, I.; Burchell, T.; Yap, G. P. A.; Richeson, D. S., Disproportionation and radical formation in the coordination of “GaI” with bis(imino)pyridines. *Dalton Transactions* **2010,** *39* (5), 1266-1272.
